# Selective CO_2_ Electroreduction to Multi-Carbon Products on Organic-Functionalized CuO Nanoparticles by Local Micro-Environment Modulation

**DOI:** 10.1007/s40820-024-01480-8

**Published:** 2024-08-08

**Authors:** Shan Ren, Xi Cao, Qikui Fan, Zhimao Yang, Fei Wang, Xin Wang, Licheng Bai, Jian Yang

**Affiliations:** 1 Materials Interfaces Center, Shenzhen Institutes of Advanced Technology, Chinese Academy of Sciences, Shenzhen, 518055 Guangdong People’s Republic of China; 2 Key Laboratory of Functional Molecular Solids, Ministry of Education, College of Chemistry and Materials Science, Anhui Normal University, Wuhu, 241002 Anhui People’s Republic of China; 3 School of Physics, Xi’an Jiaotong University, Xi’an, 710049 Shaanxi People’s Republic of China; 4 College of Materials Science and Engineering, Shenzhen University, Shenzhen, 518071 Guangdong People’s Republic of China

**Keywords:** CO_2_ electroreduction to C_2+_, Neutral electrolyte, Organic-functionalized CuO nanoparticles, Local micro-environment modulation

## Abstract

Surface functionalization of Cu-based catalysts has demonstrated promising potential for enhancing the electrochemical CO_2_ reduction reaction (CO_2_RR) toward multi-carbon (C_2+_) products, primarily by suppressing the parasitic hydrogen evolution reaction and facilitating a localized CO_2_/CO concentration at the electrode. Building upon this approach, we developed surface-functionalized catalysts with exceptional activity and selectivity for electrocatalytic CO_2_RR to C_2+_ in a neutral electrolyte. Employing CuO nanoparticles coated with hexaethynylbenzene organic molecules (HEB-CuO NPs), a remarkable C_2+_ Faradaic efficiency of nearly 90% was achieved at an unprecedented current density of 300 mA cm^−2^, and a high FE (> 80%) was maintained at a wide range of current densities (100–600 mA cm^−2^) in neutral environments using a flow cell. Furthermore, in a membrane electrode assembly (MEA) electrolyzer, 86.14% FE_C2+_ was achieved at a partial current density of 387.6 mA cm^−2^ while maintaining continuous operation for over 50 h at a current density of 200 mA cm^−2^. In-situ spectroscopy studies and molecular dynamics simulations reveal that reducing the coverage of coordinated K⋅H_2_O water increased the probability of intermediate reactants (CO) interacting with the surface, thereby promoting efficient C–C coupling and enhancing the yield of C_2+_ products. This advancement offers significant potential for optimizing local micro-environments for sustainable and highly efficient C_2+_ production.

**Supplementary Information:**

The online version contains supplementary material available at 10.1007/s40820-024-01480-8.

## Highlights

- Developing surface-functionalized catalysts with exceptional activity and selectivity for the electrocatalytic CO_2_RR to C_2+_ products under neutral electrolyte.
- A remarkable C_2+_ FE of nearly 90% at an unprecedented current density of 300 mA cm^−2^ and maintain high FE (> 80%) at the wide current density performance (100–600 mA cm^−2^) in neutral environments using a flow cell as well as MEA electrolyzer.
- Mechanical study reveals that reducing the coverage of coordinated K·H_2_O water increased the probability of intermediate reactants (CO) interacting with the surface, thereby promoting efficient C–C coupling and enhancing the yield of C_2+_ products.

**Supplementary Information:**

The online version contains supplementary material available at 10.1007/s40820-024-01480-8.

## Graphical abstract

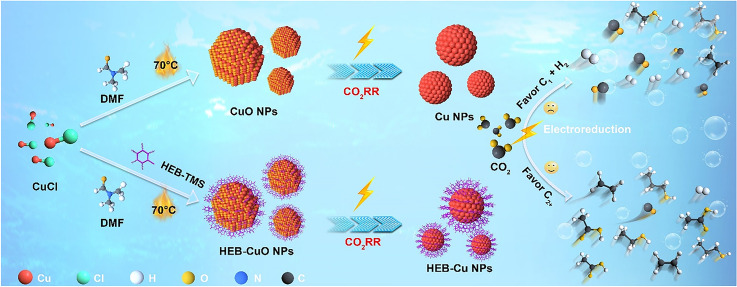

## Introduction

Anthropogenic emissions of CO_2_ resulting from the utilization of fossil fuels have exacerbated the greenhouse effect within the Earth’s atmosphere, leading to concentrations exceeding 420 ppm by 2023 and intensifying the pace at which climate change is unfolding [1, 2]. To address this ongoing warming trend, it is imperative to dedicate extensive efforts to advancing technologies for CO_2_ sequestration and conversion [3–5]. Electrochemical CO_2_ reduction (CO_2_RR) in the presence of water offers a promising avenue for driving the chemical conversion of CO_2_ by harnessing renewable electricity from sustainable sources near ambient temperature and pressure [6, 7]. In comparison with CH_4_, CO, and HCOOH, which are the major C_1_ products of the CO_2_RR [8–15], C_2+_ hydrocarbons and oxygenates (consisting of two or more carbon atoms) exhibit higher energy densities (both volumetric and gravimetric) and greater economic value, thereby experiencing higher global demand [16–19]. Compared to other catalysts, Cu-based materials demonstrate remarkable selectivity for the conversion of CO_2_ into C_2+_ products [18, 20–23]. However, the selectivity and activity of the CO_2_RR are severely limited by parasitic proton reduction (HER) occurring at similar overpotentials and high C–C coupling energy barriers [24–26]. Consequently, there is a pressing demand for the rational design of advanced Cu-based electrocatalysts capable of facilitating these processes and enabling efficient synthesis of C_2+_ products.

To achieve industrial-scale production of C_2+_, it is essential to improve CO_2_ conversion while suppressing the competing HER. Therefore, optimizing the coverage of *CO and *H on the catalyst surface should be prioritized because they are crucial indicators for predicting C–C coupling in C_2+_ production and dihydrogen evolution for H_2_ generation. However, strong electrostatic interactions resulted in a substantial increase in the adsorbed *H concentration on the catalyst surface as the applied potential increased. Consequently, this leads to a decrease in *CO coverage and impedes C_2+_ production during industrial current density electrolysis.

To address this issue, several studies have proposed diverse strategies, including regulating crystal facets [27, 28], optimizing lattice tension [29], manipulating oxidation states [8, 30], doping with heteroatoms [31], and molecule modification [32] to optimize the *CO and *H coverage balance. The surface functionalization of Cu-based catalysts not only allows for the modulation of the hydrophilic nature of the catalyst surface but also enables the optimization of the local CO_2_/H_2_O concentration, thereby influencing the surface coverage of *CO and *H, which in turn affects the reaction pathways leading to C_2+_ products. This suggests that the manipulation of micro-environmental conditions can precisely control the adsorption behavior of ions and molecules on the catalyst, thereby effectively regulating the CO_2_RR. For example, thiol-functionalized pyridine fixation on the Cu surface significantly impedes the formation of intermediates associated with CO production, thereby constraining the generation of C_2+_ products [33]. After the application of an adequate coating of polyvinylpyrrolidone polymer onto the Cu nanocrystals, complete conversion of C_2+_ to CH_4_ was observed in the CO_2_RR products, emphasizing the substantial impact of the molecular surface coverage on product selectivity [8]. However, the adopted strategies primarily focus on the electrolyzer design and optimization of the working conditions, with limited emphasis on catalyst engineering to tailor the intermediate binding energy to optimize both *CO and *H coverage. Hence, a comprehensive understanding of the adjustable *CO/*H coverage at the atomic level is imperative to achieve the CO_2_RR of C_2+_ products with an industrial-level current density.

With these motivations, we report a hexaethynylbenzene organic molecule (HEB)-modified Cu nanoparticles (HEB-Cu NPs) catalyst synthesized by in-situ electroreduction of HEB-CuO NPs under CO_2_RR conditions and further investigate the role of *CO and H* coverage in C_2+_ production. Consequently, the HEB-Cu NPs catalyst yielded a remarkable C_2+_ Faradaic efficiency (FE) of nearly 90% at an unprecedented current density of 300 mA cm^−2^ and maintained a high FE (> 80%) over a wide current density range (100–600 mA cm^−2^) in neutral environments using a flow cell. Furthermore, in a membrane electrode assembly (MEA) electrolyzer, 86.14% FE_C2+_ was achieved at a partial current density of 387.6 mA cm^−2^, while maintaining continuous operation for over 50 h at a current density of 200 mA cm^−2^, surpassing the performance of the pristine Cu NPs catalyst without HEB modification. In-situ Raman spectroscopy and molecular dynamics (MD) simulations demonstrated the substantial influence of HEB molecules on the distribution of hydrogen-bonded water coordinated at the surface of Cu NPs, increasing the coverage of *CO intermediates to further promote efficient C–C coupling and enhance the yield of C_2+_ products. The aforementioned advancement presents remarkable potential for optimizing the local micro-environment of the reaction, enabling the sustainable and highly efficient production of C_2+_.

## Experimental Section

### Materials and Chemicals

Hexakis[(trimethylsilyl)ethynyl]benzene (HEB-TMS, purity > 98%) was purchased from Zhengzhou Alfachem Co., Ltd. Copper(I) chloride (CuCl, purity > 99.99%) was purchased from Sigma-Aldrich. *N*,*N*-Dimethylformamide (DMF, AR) and ethanol absolute (C_2_H_5_OH, AR) were purchased from Sinopharm Chemical Reagent Co., Ltd. Potassium chloride (KCl, purity > 99.5%) and Nafion perfluorinated resin solution (5 wt%) were purchased from Shanghai Aladdin Biochemical Technology Co., Ltd. Potassium hydroxide (KOH, purity > 95%) was purchased from Shanghai Macklin Biochemical Technology Co., Ltd. Deuterium oxide (D_2_O, purity > 99.9 atom%D) was provided by Hebei Bailingwei Superfine Materials Co., Ltd. Cation exchange membrane (N117) and carbon paper (29 BC) were provided by Suzhou Sinero Technology Co., Ltd. All chemicals were purchased from commercial sources and used without further purification.

### Materials Characterizations

Scanning electron microscopy (SEM, HITACHI 8100) was used to examine the surface morphologies of HEB-CuO electrode. The morphology of HEB-CuO and CuO NPs was acquired by transmission electron microscopy (TEM, HITACHI HT7700). Scanning transmission electron microscopy (STEM) measurements and energy-dispersive X-ray spectroscopy (EDS) elemental mapping were taken using Talos F200S-G2. Aberration-corrected high-angle annular dark-field scanning transmission electron microscopy (AC-HAADF-STEM) was carried out on aberration-corrected JEM-ARM300F. X-ray diffraction (XRD, SmartLab 9kw, Cu-Kα (*λ* = 1.5405 Å) radiation) was adopted to characterize the crystalline structures of the synthesized NPs. The scattering range of 2*θ* was from 20° to 80° at a scanning rate of 2° min^−1^. X-ray photoelectron spectroscopy (XPS, PHI 5000 VersaProbe IV, Al Kα radiation as an exciting source) was used to determine the surface states of the HEB-CuO and CuO NPs. The Raman spectral analysis was conducted using a LabRAM HR Evolution Confocal Laser Micro-Raman spectrometer operating at a wavelength of 785 nm. ATR-SEIRAS experiments equipped with a liquid nitrogen-cooled MCT detector were performed on a Bruker INVENIO-S. X-ray absorption spectra of Cu K edges were acquired at the beamline 14 W1 of Shanghai Synchrotron Radiation Laboratory (China).

### Synthesis of HEB-CuO Nanoparticles (NPs)

10, 50, 100, and 150 mg HEB-TMS and 12 mL DMF were added in a glass bottle, respectively, to form transparent solution through ultrasonication for 5 min at room temperature, then 44.8 mg CuCl was added to the above solution and ultrasonicated for 2 min. Afterward, the bottle was sealed and heated to 70 °C in an oven for 24 h. After the reaction, the samples were centrifugated and washed with ethanol absolute. Finally, the samples were dried in a vacuum drying oven, and a black-brown powder was obtained [34].

### Synthesis of CuO NPs

44.8 mg CuCl was dissolved into 12 mL DMF in a glass bottle and ultrasonicated for 4 min (the procedure was the same). Then the above dispersion was put into a 70 °C oven for 24 h. The final product was collected by centrifugation, washed with ethanol for several times, and then dried in vacuum overnight.

## Results and Discussion

### Synthesis and Characterizations of HEB-Cu NPs Catalyst

**Fig. 1:**
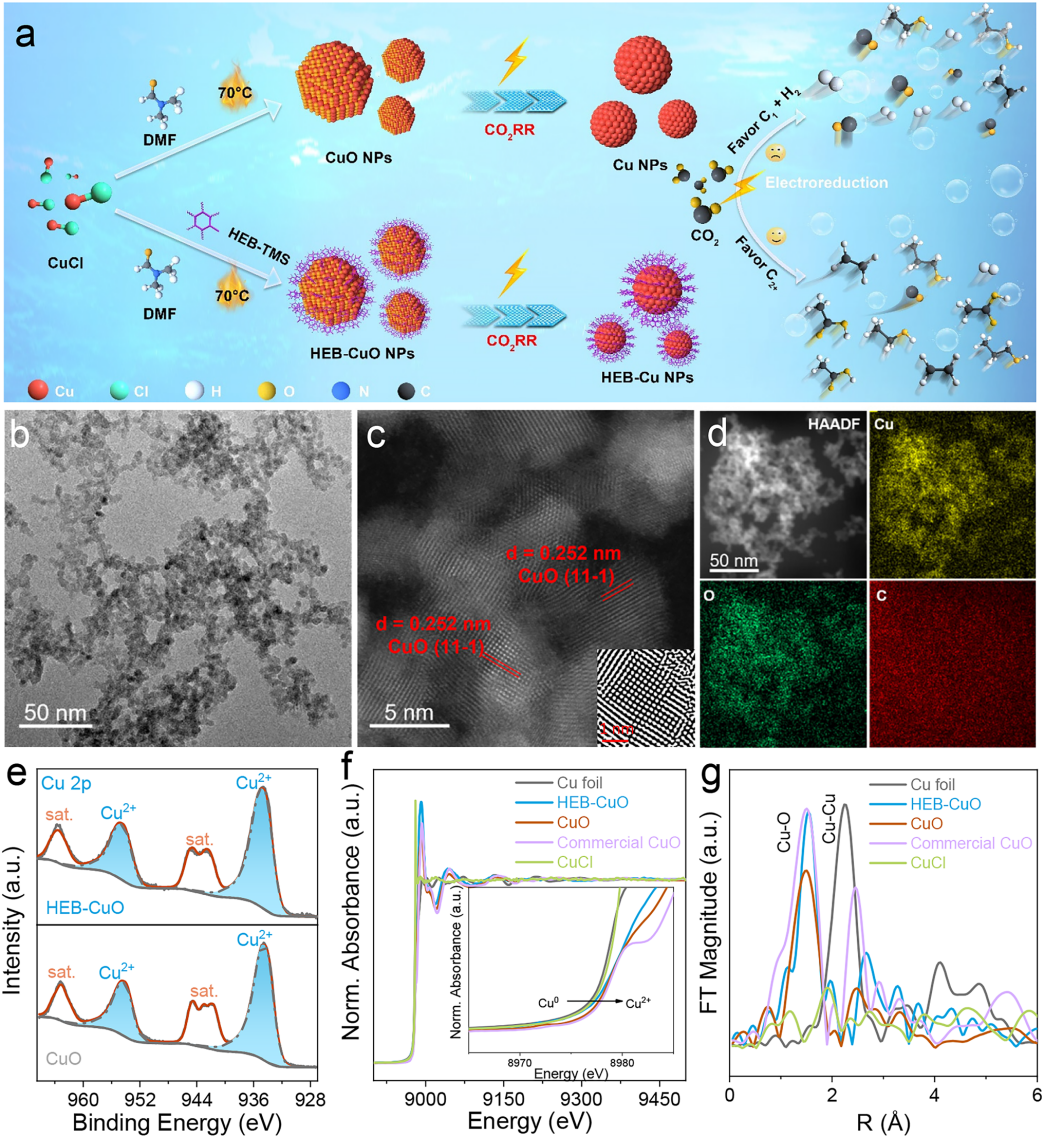
Characterization of the morphology and structure of catalysts. **a** Schematic illustration of the synthesis of HEB-CuO and CuO NPs catalysts. **b** TEM, **c** HAADF-STEM, and **d** EDS elemental mapping of HEB-CuO NPs catalyst. **e–g** Cu 2*p* XPS, K-edge XANES, and K-edge FT-EXAFS spectra of HEB-CuO and CuO NPs catalysts

Figure 1a shows the procedure for the synthesis of the HEB-Cu NPs catalyst (details in the “Supporting Information”) via the direct coupling reaction of hexakis[(trimethylsilyl)ethynyl]benzene (HEB-TMS) in *N*,*N*-dimethylformamide (DMF) using CuCl as the catalyst [34]. HEB-TMS and CuCl were added to DMF and subsequently transferred to a hermetically sealed reaction vessel for ultrasonic dispersion. After undergoing a 24-h reaction at 70 °C in the presence of oxygen, a black precipitate was formed at the bottom of the container (Fig. S1). The generated solid was recovered by centrifugation and washed consecutively with ethanol and H_2_O. The transmission electron microscopy (TEM) image in Fig. 1b illustrates a well-organized and uniformly spherical structure measuring approximately 5 nm in diameter, which is consistent with previous findings [34]. The XRD pattern demonstrates that the HEB-CuO exhibits a crystal phase, which can be assigned to the monoclinic CuO (space group = *C2/c*) with *a* × *b* × *c* = 4.688 × 3.423 × 5.132 Å^3^, in comparison with that of the precursor of CuCl, providing strong evidence for the formation of a new arrangement (Fig. S2). The lattice fringe distance obtained from the high-angle annular dark-field scanning TEM (HAADF-STEM) image was 0.252 nm, corresponding to the (11-1) planes of monoclinic CuO (Fig. 1c), in agreement with the XRD results. The EDS results revealed the presence of Cu, O, and C throughout the nanoparticles (Fig. 1d). Because EDS mapping lacks sensitivity in detecting light elements, the presence of HEB molecules on the surface of CuO NPs was further confirmed through electron energy loss spectroscopy (EELS) characterization. Compared to the CuO NPs synthesized without the addition of the HEB (Fig. S3), the HEB-CuO catalyst exhibited a distinct carbon peak at 288 eV, which corresponded to the presence of HEB molecule (Fig. S4). Meanwhile, the presence of the HEB molecule on the surface of CuO NPs was further confirmed through Fourier transform infrared (FTIR) spectroscopy, as shown in Fig. S5. Specifically, the characteristic peaks observed at 1400–1500, 1500–1600, 893, and 3068 cm^−1^ are attributed to the asymmetric stretching mode of the C–C bonds, skeletal vibrations of the aromatic ring, and bending and stretching vibrations of the aromatic C–H bonds, respectively [35, 36]. The XPS is further employed to study the surface elemental states of the Cu species. As shown in Fig. 1e, the Cu 2*p*_3/2_ peak observed at a binding energy of approximately 934.6 eV in the HEB-CuO can be attributed to the presence of Cu^2+^ [8], which is analogous to that detected in CuO. Moreover, X-ray absorption near-edge structure (XANES) and extended X-ray absorption fine structure (EXAFS) were utilized to better evaluate the electronic state of Cu in the CuO and HEB-CuO catalysts. The absorption spectrum of the Cu K-edge was observed (Fig. 1f), with the Cu electronic state of HEB-CuO falling between that of the Cu foil and commercial CuO, while the Cu electronic state of HEB-CuO approached that of CuO. The Fourier transform (FT) EXAFS spectrum in Fig. 1g also displays similar characteristic peaks of CuO and HEB-CuO in the R-space, mainly in the form of Cu–O bonds. These results indicate that HEB modification had no discernible impact on the electronic structure of the CuO surface.

### Catalytic Performance of CO_2_RR in Flow Cell

**Fig. 2:**
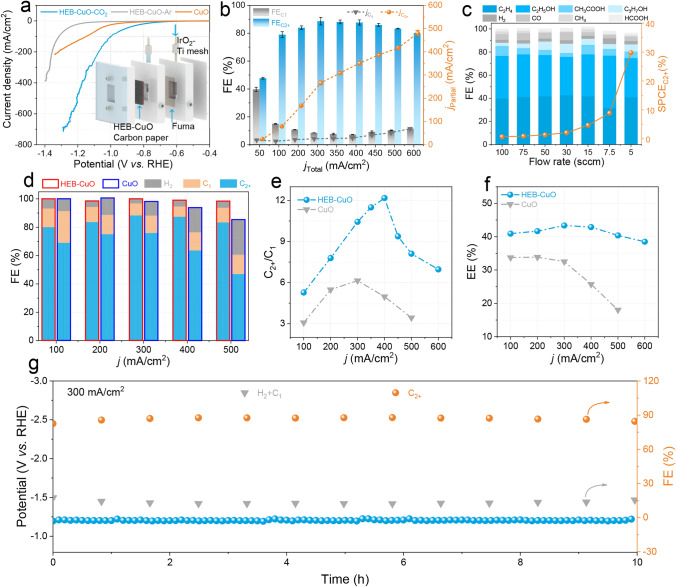
Electrocatalytic CO_2_RR performance in a flow cell. **a** LSV curves of HEB-CuO and CuO NPs in CO_2_ and Ar atmospheres. **b** FE and partial current density of C_1_ and C_2+_ products at different current densities. **c** CO_2_RR-based ${\text{SPCE}}_{{{\text{C}}_{{{2} + }} }}$ versus CO_2_ flow rate performance of HEB-CuO catalyst in 1M KCl solution. **d** FE of H_2_, C_1_, and C_2+_. **e** C_2+_/C_1_ selectivity and **f** the half-cell energy efficiency of C_2+_ products for HEB-CuO and CuO NPs. **g** Stability of HEB-CuO at 300 mA cm^−2^ in 1M KCl solution

Considering the experimentally and theoretically verified high CO_2_RR activity showcased by Cu-based materials, our study aimed to investigate the activity and selectivity for the neutral condition of HEB-CuO (optimal concentration of HEB-TMS, Fig. S6) and CuO NPs electrocatalysts, which are painted onto the hydrophobic gas diffusion layer carbon paper (Sigracet 29BC) with a thickness of approximately 10 μm (Fig. S7) in a flow cell configuration (Fig. S8). The cathode was equipped with a peristaltic pump, while the anode was fitted with a gas–liquid mixed flow pump to ensure consistent electrolyte pH and optimal electrode contact. This configuration facilitates the efficient removal of liquid or gas byproducts while promoting CO_2_ gas reactions at the triple-phase boundaries within the cell device. Hence, after activation of the electrocatalyst, representative polarization curves for HEB-CuO and CuO were obtained in a neutral 1M KCl electrolyte under different atmospheres (Figs. 2a, S9). The HEB-CuO electrocatalyst exhibited a significantly enhanced current–voltage response in the presence of CO_2_ compared to Ar, with a lower onset potential and higher current density. To improve the performance, a collaborative assessment was conducted on the HEB-CuO and CuO NPs. As shown in the linear scanning voltammeter (LSV) curves (Fig. 2a), the HEB-CuO electrocatalyst has lower onset potentials at ~ − 0.7 V in neutral electrolyte than that of CuO (~ −0.9 V), demonstrating that the surface modification of CuO catalyst with HEB organic molecule can significantly reduce the overpotential required for CO_2_ activation. To ensure a more accurate analysis of the FE for all gas and liquid CO_2_RR products, it is crucial to employ real-time monitoring of the outlet flow rate using a Defender 520 flow meter. Both the catholyte and anolyte were collected for ^1^H nuclear magnetic resonance (NMR) analysis. Gas chromatography (Shimadzu 2014C) and liquid ^1^H NMR spectroscopy (Bruker 400 M) enabled us to obtain representative patterns that exclusively detected H_2_, C_1_ (CO, CH_4_, and HCOOH), and C_2+_ (C_2_H_4_, C_2_H_5_OH, CH_3_COOH, and C_3_H_7_OH) products in the gas and liquid phases (Fig. S10). The collection time for each product at different current densities was no less than 1000 s (Fig. S11), to ensure data accuracy. As shown in Figs. 2b, S12, and Table S1, the selectivity of HEB-CuO toward C_2+_ exhibited an increasing trend with progressive augmentation in current density, while the selectivity toward H_2_ and C_1_ demonstrated a decreasing tendency. A high plateau of C_2+_ selectivity > 80% was consistently maintained over a wide current density range of 100–600 mA cm^−2^ with a maximum C_2+_ selectivity of 88.62% at 300 mA cm^−2^ in a neutral solution, and the counter HER and C_1_ were suppressed below 12%. Additionally, HEB-CuO achieves a maximum $j_{{{\text{C}}_{{{2} + }} }}$ up to 480.5 mA cm^−2^ at a ${\text{FE}}_{{{\text{C}}_{{{2} + }} }}$ of nearly 80%, which is far surpassing the industrial current density requirements for CO_2_RR to C_2+_ (> 200 mA cm^−2^). Figure 2c presents the single-pass carbon efficiency (SPCE) of the C_2+_ products at different CO_2_ flow rates measured at 300 mA cm^−2^. At higher flow rates, the SPCE was lower owing to the excessive CO_2_ input compared to its consumption. By gradually lowering the CO_2_ flow rate from 100 to 5 sccm, the SPCE achieved a high value of ~ 1% to ~ 30% for C_2+_ production under neutral conditions. Through gas-phase exchange experiments (CO_2_/N_2_), we further validated that the formation of C_2+_ products resulted from CO_2_ molecules rather than the decomposition of the ligand (Fig. S13). The FE of the C_2+_ products for the CuO electrocatalyst was evaluated under the same conditions with that of HEB-CuO (Figs. 2d, S14). The performance of CO_2_RR to C_2+_ exhibited by the CuO electrocatalyst is deemed unsatisfactory, particularly with regard to the significant H_2_ evolution observed at higher current densities. The electrochemical impedance spectra (EIS) of HEB-CuO (Fig. S15) exhibited the fastest interface charge-transfer rate, which was beneficial for enhancing the reaction rate. Normalization of partial current densities for reduced C_2+_ products is conducted using the electrochemical active surface area (ECSA) (Fig. S16). HEB-CuO displayed a greater C_2+_ current density normalized by the ECSA value than CuO, indicating higher intrinsic activity toward C_2+_ products. In Fig. S17, HEB-CuO showed a consistent OH_ad_ peak with CuO but with a much stronger OH^−^ characteristic peak, which indicates that the local alkalinity of HEB-CuO increased and contributed to the improvement of C–C coupling [37, 38]. Moreover, the encapsulation of HEB by CuO effectively improved the selectivity for C_2+_ products, with a C_2+_/C_1_ ratio of 12.2, which is 2.5 times of the bare CuO NPs at a current density of 400 mA cm^−2^ (Fig. 2e). Furthermore, the energy efficiency of the half-cell (EE_half-cell_) was calculated based on the cathode (CO_2_RR) and anode (oxygen evolution reaction) processes. A high plateau of the EE_half-cell_ of HEB-CuO was consistently maintained at > 40% over a wide current density range of 100–500 mA cm^−2^ with a maximum EE_half-cell_ of 43.3% at 300 mA cm^−2^, which was much higher than that of CuO without HEB modification (Fig. 2f). These findings unequivocally illustrate the pivotal contribution of the HEB molecules in significantly lowering the activation energy barrier for CO_2_ and efficiently inhibiting the generation of related byproducts, particularly with regard to attenuating the HER at high current densities. Notably, the HEB-CuO electrocatalyst demonstrated exceptional long-term durability in a flow cell, maintaining a high current density (300 mA cm^−2^ in a neutral electrolyte) throughout 10 h of continuous operation with only a marginal decrease in FE_C2+_, while the FE of H_2_ and C_1_ remained stable (Fig. 2g). This discovery holds significant importance in the field of electrocatalytic CO_2_RR.

### Catalytic Performance of CO_2_RR in MEA

**Fig. 3:**
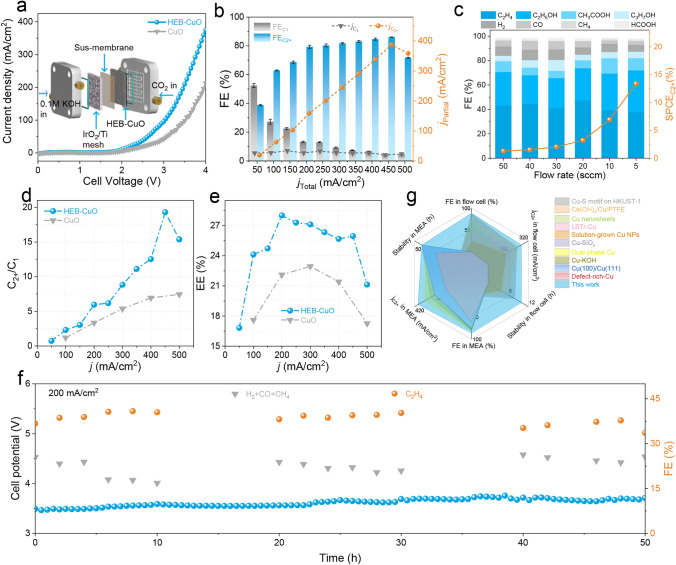
Electrocatalytic CO_2_RR performance in catholyte-free MEA electrolyzer. **a** Current densities versus applied voltages between 0 and 4 V for HEB-CuO and CuO NPs in CO_2_ atmospheres. **b** FE and partial current density of C_1_ and C_2+_ products at different current densities and **c** ${\text{SPCE}}_{{{\text{C}}_{{{2} + }} }}$ versus CO_2_ flow rate performance of HEB-CuO electrocatalyst. **d** C_2+_/C_1_ selectivity and **e** the full-cell energy efficiency of C_2+_ products for HEB-CuO and CuO NPs electrocatalysts. **f** CO_2_RR stability on HEB-CuO at 200 mA cm^−2^. **g** Comparison of the HEB-CuO with the reported state-of-the-art Cu-based catalysts. All comparison data points are from the references summarized in Table S3

Considering the voltage drop induced by the internal resistance of the solution, particularly at higher kinetic current densities, resulting in a significant decrease in energy efficiency and gas diffusion layer flooding in the flow cell configuration, we further employed an anion MEA gas-phase electrochemical electrolyzer with a 1 cm^2^ active area to investigate the performance of the CO_2_RR. The cathode side was supplied with humidified CO_2_ gas to prevent direct contact between the catalysts and liquid water, thereby limiting reactant diffusion. Additionally, a 0.1M KOH solution was circulated on the anode side. The structure and assembly processes are shown in Fig. S18. With a gradual increase in the cell voltage (Fig. 3a), the total current density of the HEB-CuO electrocatalyst exhibited a progressive enhancement, ultimately peaking at 400 mA cm^−2^ without iR compensation, significantly surpassing that of CuO. This observation is consistent with the flow cell configuration. More importantly, HEB-CuO exhibits C_2+_ product selectivity with a trend similar to that of the flow cell, effectively suppressing C_1_ and H_2_ generation. It maintained a low FE for the H_2_ and C_1_ products while achieving FE_C2+_ above 80% over a wide range of current densities from 200 to 450 mA cm^−2^ (Figs. 3b, S19, and Table S2). Additionally, HEB-CuO exhibits a peak *j*_C2+_ of up to 450 mA cm^−2^ when FE_C2+_ reaches 86.14%, which far surpasses that of the CuO electrocatalyst for the CO_2_RR to C_2+_ (Fig. S20). Figure 3c shows the FE and SPCE of C_2+_ with respect to the CO_2_ gas flow rate. The C_2+_ FE could be maintained at a high level, whereas the CO_2_ flow rate persistently declined. The highest SPCE of 13.4% for the C_2+_ products was achieved at 5 sccm, indicating the enormous potential of HEB-CuO for CO_2_RR performance. The C_2+_/C_1_ ratio for HEB-CuO was 19.2, which was 2.7 times that of CuO at a current density of 450 mA cm^−2^ (Fig. 3d). Moreover, the full-cell energy efficiency EE_full-cell_ of the C_2+_ products calculated at different current densities was higher than that of CuO (Fig. 3e), further demonstrating that HEB molecule modification can significantly lower the activation energy barrier for CO_2_ and efficiently inhibit the generation of related byproducts. Achieving long-term operational stability is a key requirement for practical applications but still presents challenges. Therefore, the stability of CO_2_ electrolysis over the HEB-CuO catalyst was further measured at a fixed applied current density of 200 mA cm^−2^ using a 0.1M KOH anolyte (Fig. 3f). The electrolyzer maintained a stable full-cell potential with ${\text{FE}}_{{{\text{C}}_{{2}} {\text{H}}_{{4}} }}$ and ${\text{FE}}_{{({\text{CO}} + {\text{CH}}_{{4}} + {\text{H}}_{{2}} )}}$ exhibiting complementary trends. Continuous operation for over 50 h was achieved at 200 mA cm^−2^ with steady ${\text{FE}}_{{{\text{C}}_{{2}} {\text{H}}_{{4}} }}$ levels. These results indicate that HEB-CuO demonstrates excellent activity for the electrocatalytic CO_2_RR of C_2+_ products in both neutral flow cell and MEA (Fig. 3g).

### Discussions

**Fig. 4:**
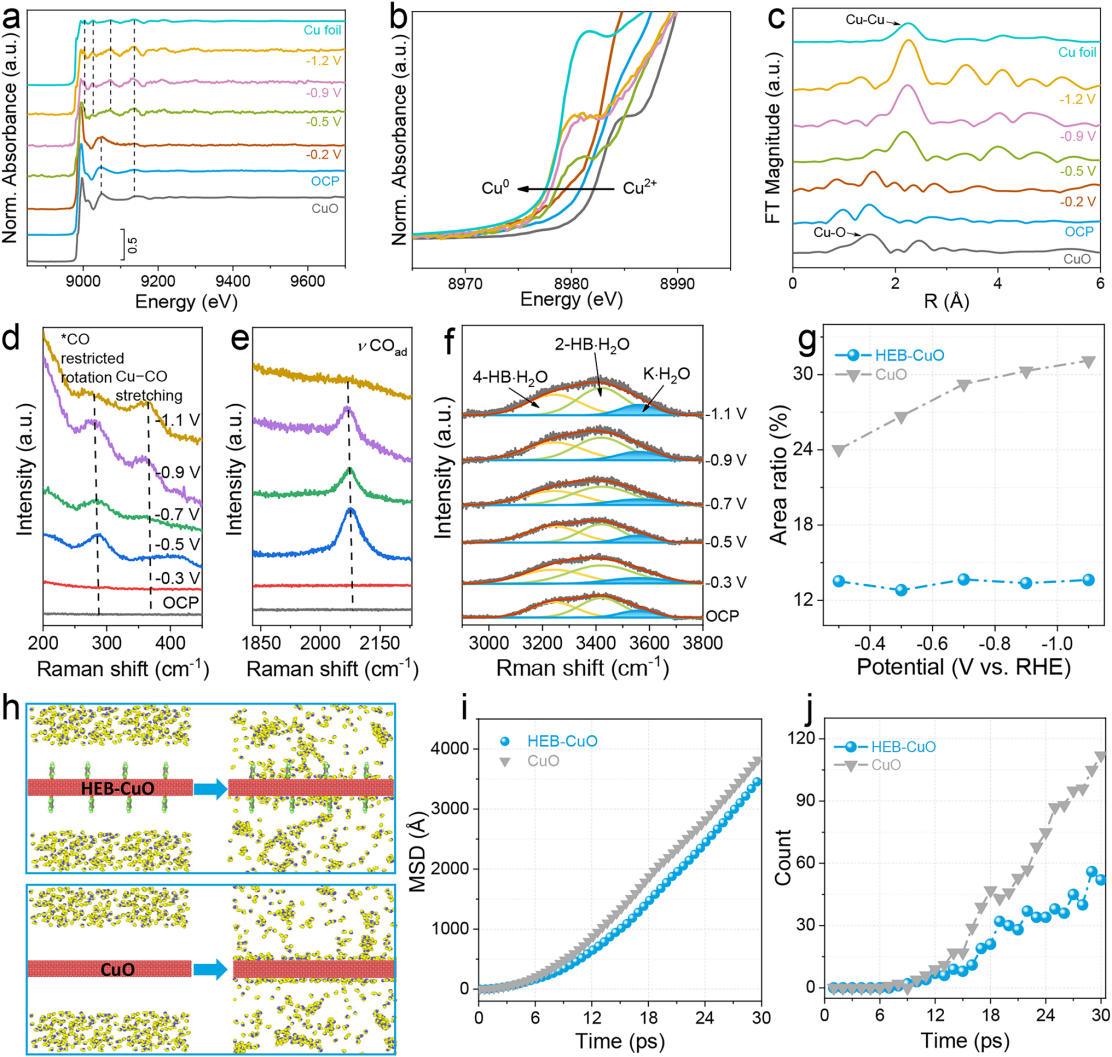
Mechanism investigation on the catalytic activity. **a** In-situ XANES spectra at the Cu K-edge. **b** Details of in-situ XANES spectra and **c** in-situ FT-EXAFS spectra in R-space of the HEB-CuO catalyst during CO_2_RR. **d**, **e** In-situ Raman spectra at different ranges of the HEB-CuO catalyst. **f** In-situ Raman spectra of the interfacial water structure on HEB-CuO from OCP to − 1.1 V. **g** Quantification of the area percentages of K·H_2_O peaks observed at different applied potentials on HEB-CuO and CuO electrocatalysts. **h** Molecular dynamics simulation snapshots depicting the diffusion of H_2_O molecules in the vicinity of a Cu surface, subject to varying levels of stress at 30 ps. **i** Analysis of the mean square displacements of H_2_O at the Cu surface of HEB-CuO and CuO. **j** Investigation of the number of surface H_2_O observed near the Cu surface at varying strain levels of HEB-CuO and CuO

To facilitate a more comprehensive investigation into the in-situ evolution of the derived structures and the underlying mechanism of the electrocatalytic CO_2_RR, relevant ex-situ and in-situ characterizations were conducted. As shown in Figs. S21 and S22, the HEB-CuO and CuO electrocatalysts after the CO_2_RR did not change significantly in morphology, suggesting their structural stability. The EELS mapping and spectra of HEB-CuO after the CO_2_RR tests (Fig. S23) also exhibited a carbon peak at 288 eV with that of the HEB-CuO catalyst, corresponding to the presence of the HEB molecule. Meanwhile, the HRTEM images revealed the presence of the CuO crystal phase, while preliminary color observations following the reaction indicated the formation of metallic Cu, suggesting a reduction in CuO (Fig. S24). The surface of the reacted gas diffusion electrode (GDE) was characterized by XPS. The Cu 2*p* spectrum shows that Cu^0^ exists after the reaction (Fig. S25), confirming that Cu(II) was reduced to Cu^0^. Further analysis of the Cu LMM Auger spectra (Fig. S26) shows that the Cu^2+^ peak shifted significantly to the Cu^0^ peak after the reaction [39]. Furthermore, the in-situ XANES spectra were obtained in neutral media to validate the phase structure transformation process at various potentials (Figs. 4a, S27). Examination of both near-edge and extended-edge structures revealed a progressive decline in the oxidation state of the catalyst from Cu^2+^ to Cu^0^ with increasing potential (Fig. 4b). Fourier transform analysis revealed a gradual increase in the strength of Cu–Cu coordination, while the intensity of Cu–O coordination decreased, indicating that Cu^2+^ was completely reduced to metal Cu species, which fully agrees with the color changes. Fourier transform analysis demonstrated a progressive enhancement in the strength of Cu–Cu coordination, accompanied by a decrease in the intensity of Cu–O coordination, suggesting the complete reduction of Cu^2+^ to metallic Cu species (Fig. 4c). These findings are consistent with the observed color alterations. To gain insight into the key intermediates of electrocatalysts for CO_2_RR to C_2+_ products, we performed in-situ Raman spectroscopy with a flow cell in KCl electrolyte (Fig. S28). Starting at the open-circuit potential (OCP), the Raman spectra of the HEB-CuO and CuO catalysts show several peaks (Figs. 4d, e, and S29). The peaks at 280, 365, and 2080 cm^−1^ correspond to the stretching mode of Cu–CO and the atop configuration of *CO adsorption, respectively, which are widely employed to assess the *CO coverage on a catalyst [40, 41]. As shown in Figs. 4d and S29a, the HEB-CuO catalyst appears the obvious Cu–CO restricted rotation and stretching modes at a more positive potential − 0.5 V, demonstrating the modification of HEB enables the acceleration of reaction kinetics for CO_2_RR to *CO intermediate. Moreover, in contrast to the absence of a distinct *CO adsorption peak at atop sites of CuO, a newly emerged atop-adsorbed *CO species is observed on HEB-CuO over a wide potential range from OCP to − 0.9 V, providing further evidence for an enhanced *CO coverage (Figs. 4e, S29b). To gain further molecular-level insight into the *H intermediate of the catalysts, the characteristic peaks of interfacial water at approximately 3400 cm^−1^ were classified into three distinct components: 4-HB·H_2_O, representing water with four coordinated hydrogen bonds at approximately 3240 cm^−1^; 2-HB·H_2_O, representing water with two coordinated hydrogen bonds at approximately 3420 cm^−1^; and K·H_2_O, representing water hydrated by K^+^ ions at approximately 3555 cm^−1^ (Figs. 4f, S30) [32, 42, 43]. The percentage of the K·H_2_O peak generally increased at higher applied negative potentials, demonstrating a decrease in hydrogen bonding and an increase in *H coverage. Upon increasing the applied potential from OCP to − 1.1 V, the proportion of K·H_2_O on the pristine CuO surface significantly increased from 24.0% to 31.1%, while only exhibiting a marginal change from 12.8% to 13.6% on HEB-CuO (Fig. 4g). This observation implies that pristine CuO exhibits pronounced efficacy for water dissociation and *H adsorption. Considering the divergent trends observed for *CO over HEB-CuO and CuO under increasing cathodic bias, it can be inferred from Fig. 4d, e that the enhanced CO_2_RR to C_2+_ activity exhibited by HEB-CuO compared to that of CuO is likely due to the reduced presence of interfacial water (K·H_2_O). Moreover, the adsorption of water molecules on the HEB-CuO and CuO electrocatalysts during the CO_2_RR was further investigated via MD simulations conducted in a canonical ensemble at 300 K for a duration of 30 ps (Fig. 4h). The Mean Square Displacement (MSD) of water molecules, which serves as an indicator of their mobility, exhibited a declining trend in the presence of the HEB organic molecules coated on the CuO surface (Fig. 4i). Furthermore, the number of water molecules for HEB-CuO showed a significant decrease over time compared with that of CuO electrocatalyst (Fig. 4j). These results provide strong evidence supporting the efficient repulsion of water molecules from the Cu catalyst by the HEB organic molecules, which is consistent with the findings obtained through in-situ Raman spectroscopy. Meanwhile, the in-situ attenuated total reflection surface-enhanced infrared spectroscopy (ATR-SEIRAS) (Figs. S31, S32) demonstrates the enhanced clarity of the peaks at 1092 and 1415 cm^−1^, attributed to *CO*COH and *CO*CO on HEB-CuO compared to pristine CuO, indicating a significantly higher *CO coverage on the electrode surface [44]. This pronounced increase in the C–C coupling probability results in the increased generation of C_2+_ products. Consequently, the HEB-CuO catalyst infers superior FE_C2+_ values across a wide range of current densities compared to CuO catalysts, which can be ascribed to both limited *H coverage and an elevated CO to H_2_O ratio (Figs. 2b, 3b).

## Conclusions

In conclusion, we developed a surface HEB molecule functionalization method for CuO NPs electrocatalyst, wherein the optimization of local micro-environment modulation, particularly with respect to the surface coverage of *CO and *H, influences the reaction pathways leading to C_2+_ products. This approach enables the simultaneous achievement of a high proportion of *CO and a low concentration of *H, thereby facilitating the efficient conversion of CO_2_ to C_2+_ products. The HEB-modified CuO NPs catalyst exhibits outstanding FE_C2+_ of nearly 90% at an unprecedented current density of 300 mA cm^−2^ and maintains high FE (> 80%) at the wide current density performance (100 to 600 mA cm^−2^) in neutral environments using a flow cell and 86.14% FE_C2+_ at a partial current density of 387.6 mA cm^−2^, in a MEA electrolyzer. The current study not only provides comprehensive insights into the influence of *CO and *H coverage on C–C coupling but also lays the foundation for catalyst engineering toward achieving industrial-level CO_2_ electrolysis in practical MEA devices.

## Supplementary Information

Below is the link to the electronic supplementary material.

Supplementary file1 (PDF 3330 KB)
