# Supplementary material for: Selective CO2 Electroreduction to Multi-Carbon Products on Organic-Functionalized CuO Nanoparticles by Local Micro-Environment Modulation
Source: Nanomicro Lett. 2024 Aug 8;16:262. doi: 10.1007/s40820-024-01480-8 (PMC11310183; doi:10.1007/s40820-024-01480-8)
Supplement: Supplementary file 1 — Supplementary file1 (PDF 3330 KB) [file 40820_2024_1480_MOESM1_ESM.pdf]

Supporting Information for

Selective CO_2 Electroreduction to Multi-Carbon Products on Organic-Functionalised CuO Nanoparticles by Local Microenvironment Modulation

Shan Ren[1], Xi Cao[2], Qikui Fan[3, *], Zhimao Yang[3], Fei Wang[1], Xin Wang[1], Licheng Bai[1, *], Jian Yang[2, 4, *]

[1] Materials Interfaces Center, Shenzhen Institutes of Advanced Technology, Chinese Academy of Sciences. Shenzhen, Guangdong 518055, P. R. China

[2] Key Laboratory of Functional Molecular Solids, Ministry of Education, College of Chemistry and Materials Science, Anhui Normal University. Wuhu, Anhui 241002, P. R. China

[3] School of Physics, Xi'an Jiaotong University. Xi'an, Shaanxi 710049, P. R. China

[4] College of Materials Science and Engineering, Shenzhen University. Shenzhen, Guangdong 518071, P. R. China

*Corresponding authors. E-mail: fanqikui@xjtu.edu.cn (Qi-Kui Fan); licheng_bai@163.com (Li-Cheng Bai); yang520@mail.ustc.edu.cn (Jian Yang)

S1 Experimental Section

S1.1 Electrocatalytic CO_2 reduction

Electrode Preparation. In brief, 5.0 mg HEB-CuO catalyst and 50 μL Nafion were added to solvent with 1 mL deionized water and ethanol (in a mass ratio of 1:1) to form an ink by ultrasonication for 10 min. For CuO catalyst, the same combination of Nafion and deionized water and ethanol solvent should be used. The obtained ink was then sprayed onto a gas-diffusion carbon papers with areas of 0.5 × 2 cm^2 (for flow cell), 1×1 cm^2 (for MEA) by using an airbrush. Subsequently, the obtained gas-diffusion electrodes (GDEs) were dried under vacuum conditions for CO_2RR tests.

CO_2RR Test. The electrochemical measurements were conducted under room temperature and pressure conditions using a CHI electrochemical workstation (1140C series). CO_2RR tests were performed in both the flow cell and MEA, with each test maintained for 25 minutes at various current densities. The cathode was the prepared GDEs while an IrO_x-coated Ti mesh served as the anode. CO_2 gas was passed through the flow cell and MEA gas chamber at different flow rates controlled by a mass flow controller. Both gas-phase and liquid-phase data reported in this study underwent error correction based on three independent measurements.

For the electrochemical test in a flow cell, the three-electrode configuration was employed with iR correction. All applied potentials were measured against an Ag/AgCl reference electrode (saturated KCl) and converted to the RHE reference scale using the following equation: E (vs. RHE) = E (vs. Ag/AgCl) + 0.197 + (0.0591 ∗ pH) - iR. The solution resistance of the electrolyte, denoted as R, was determined through electrochemical impedance spectroscopy test. A cation exchange membrane (N117) separated the cathodic chamber and the anodic chamber. Peristaltic pumps circulated 1 M KCl (KOH) and KOH aqueous solutions through the cathodic and anodic chambers at a rate of 5 mL/min, respectively. For the stability test, new 1 M KCl and 1 M KOH solutions were periodically introduced into both catholyte and anolyte compartments to restore their respective ionic concentrations and conductivities.

For the electrochemical tests in MEA, a Sustainion anion-exchange membrane was used to separate the cathodic chamber and anodic chamber. CO_2 gas at varying feed rates was continuously supplied to the humidifier with distilled water and then introduced into the cathode

chamber. A flow of 0.1 M KOH solution at a rate of 5 mL/min was introduced into the anode chamber. The performance evaluation of the cathode electrode was conducted in a two-electrode system under different current densities. The products generated on the cathode side were passed through a cold trap for separation into liquid and gas phases. To ensure stability, periodic replacement of the anolyte with fresh 0.01 M KOH solution was carried out to restore ionic concentration and conductivity.

The gaseous products were collected using a gas bag and quantified by gas chromatography (GC-2014, Shimadzu), equipped with thermal conductivity detector (TCD), utilizing nitrogen as the carrier gas. The faradaic efficiency (FE) of each gaseous product i was determined using the following equation S1:

$$FE_i = \frac{Q_i}{Q_{total}} \times 100\% = \frac{n_i \times C_i \times v \times t \times F}{I \times t \times V_M} = \frac{n \times C_x \times v \times F}{I \times V_M} \quad (S1)$$

where FE_i is the faradaic efficiency of the gas product i, Q_i is the charge of the product i formation, Q_{total} is the total charges passed through the working electrode, n_i is the amount of electron transfer for reduction to the molecular product i, C_i is the volume fraction of product i detected by GC, v is the outlet gas flow rate, t is the CO_2 electrolysis time, F is the Faradaic constant (96,485 C/mol), I is the total current during CO_2 electrolysis, and V_M is the gas molar volume at room temperature (24.5 L/mol).

The liquid product was collected from anode, cathode and gas sides and analyzed by 1H NMR (Bruker Advance III 400 HD spectrometer). A collection solution of 500 μL was mixed with 200 μL D_2O containing 50 ppm (m/m) dimethyl sulfoxide (DMSO) as an internal standard. Identical spectral acquisition parameters were used for all measurements to ensure complete relaxation and quantification.

$$FE_i = \frac{Q_i}{Q_{total}} \times 100\% = \frac{n_i \times C_i \times V \times F}{I \times t} \quad (S2)$$

where FE_i is the faradaic efficiency for the liquid product i, Q_i is the charge of product i formation, Q_{total} is the total charge passed through the working electrode, n_i is the amount of electron transfer for reduction to the molecule product i, C_i is the concentration of the product i in the catholyte gained by NMR, V is the volume of the catholyte, t is the CO_2 electrolysis time, F is the Faradaic constant (96,485 C/mol), I is the total current during CO_2 electrolysis, t is the CO_2 electrolysis time.

The half-cell (cathodic) energy efficiency ($EE_{half\text{-}cell}$) of the i product was calculated as follows:

$$EE_{half-cell_{C2+}} (\%) = \sum \frac{(1.23 - E_i) \times FE_i}{1.23 - E} \quad (S3)$$

where E_i is the thermodynamic potential for product i [S1], E is the applied potential vs. RHE and FE_i is measured Faradaic efficiency for product i.

The full-cell energy efficiency ($EE_{half\text{-}cell}$) of the i product was calculated by the following equation S4:

$$EE_{full-cell_{C2+}} (\%) = \sum \frac{(1.23 - E_i) \times FE_i}{-E_{full-cell}} \quad (S4)$$

where E_i is the thermodynamic potential of product i, FE_i is measured faradaic efficiency of product i, $E_{full\text{-}cell}$ is the full-cell voltage measured in the MEA system without ohmic loss correction.

Single-pass carbon efficiency (SPCE) towards products was calculated as follows:

$$SPCE_{C2+} = \sum \frac{j \times 60s \times FE_i}{v \times 1min} \times V_M \quad (S5)$$

where FE_i is measured faradaic efficiency of product i, j is the partial current density, v is the measured flow rate, V_M is the molar volume of the gas (22.4 L/mol).

The formation rate (R) for each species (i) was calculated by the following equation S6:

$$R_{C2+} = \sum \frac{Q_{total} \times FE_i}{F \times Z_i \times t \times S} \tag{S6}$$

Where FE_i is measured Faradaic efficiency for product i, Q_{total} is the total charge passed through the working electrode, F is the Faradaic constant (96,485 C/mol), Z_i is the number of electrons required to produce one molecule of product, t is the electrolysis time, S is the geometric area of the electrode.

S1.2 *In situ* spectroscopy

***In-situ* ATR-SEIRAS measurement.** The measurements were carried out using a three-electrode configuration which Pt foil and a saturated Ag/AgCl as the counter electrode and reference electrode, respectively. The working electrode was prepared by depositing a gold film onto silicon and subsequently dropping catalyst ink onto it. The catholyte was 1 M CO_2-saturated KCl electrolyte while the anolyte was 1 M KOH. The spectrums were *in situ* collected under open circuit potential (OCP) and different ALPS electrolysis steps.

***In-situ* Raman spectroscopy.** *In-situ* Raman spectra was performed using a Raman flow cell with a 785 nm excitation laser. During the experiment, 1 M KCl and KOH aqueous were passed through the cathodic chamber and anodic chamber, respectively. The electrolyte was circulated at a rate of 5 mL/min using peristaltic pumps. The flow rate of CO_2 was kept at 100 sccm with a mass flow controller.

***In-situ* XAS measurement.** The measurements were conducted for Cu K-edge absorption at BL11B-XAFS Beamline in Shanghai Synchrotron Radiation Facility, China. Total-fluorescence-yield mode was employed to record the corresponding data. The *in-situ* XAS fluorescence cell utilized a three-electrode setup with graphite rod and saturated Ag/AgCl as the counter electrode and reference electrode, respectively. The working electrodes were prepared by dropping catalyst ink onto carbon paper substrate. The 1 M KCl electrolyte continuously received high purity (99.999%) CO_2 gas supply.

S1.3 Molecular dynamics (MD) simulation

Molecular dynamics (MD) [S2] simulations are computational methods used to simulate and study the motion of atoms or molecules over time and space. Using MS software, a spatial model of Cu-loaded ligands with H_2O was established and optimized. The simulation box size is 108.94×54.72×141.67 Å, containing 6619 Cu atoms, 582 water molecules, and 16 ligands. Each ligand consists of 18 carbon atoms, 5 hydrogen atoms, and 1 Cu atom. The bulk Cu is located at the center of the simulation box, with the Cu and substrate interface oriented at [001]. The dimensions of the Cu model are 1084.94×54.22×10.84 Å. Water molecules are randomly distributed in the simulation box to mimic the liquid state of water, and the copper substrate's center of mass is fixed to stabilize the Cu. The consistent valence force-field (CVFF) was used for phenol [S3]. The interaction potentials between oxygen atoms and between Cu atoms and oxygen atoms in water molecules were described by the Lennard-Jones (L-J) potential, ignoring the interaction potentials among hydrogen atoms and between them and other atoms. The Lennard-Jones (L-J) potential is expressed as equation S7 [S4]:

$$\phi(r) = 4\varepsilon\left[\left(\frac{\sigma}{r}\right)^{12} - \left(\frac{\sigma}{r}\right)^{6}\right] \tag{S7}$$

where $\varphi(r)$ is the potential energy between a pair of atoms, r is the separation distance, ε is the depth of the potential well, and σ is the Van der Waals separation distance. The canonical

ensemble (NVT) [S5] simulations were carried out at constant temperature (300 K) with a Nose-Hoover thermostat [S6] for 30 ps with a time step of 1 fs.

S2 Supplementary Figures and Tables

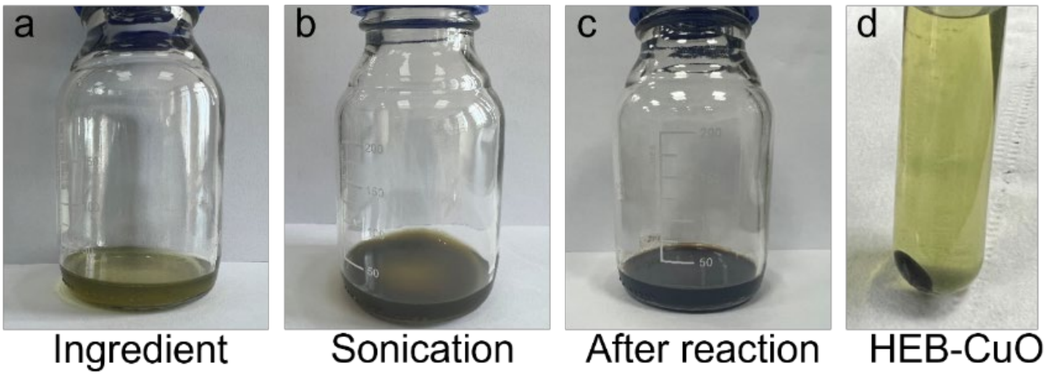

Fig. S1 Digital images of HEB-CuO catalyst synthesis. (**a**) HEB-TMS and CuCl are mixed with DMF. (**b**) Ultrasonication for 2 min. (**c**) Heated in 70 °C oven for 24 h. (**d**) Centrifugation and washed

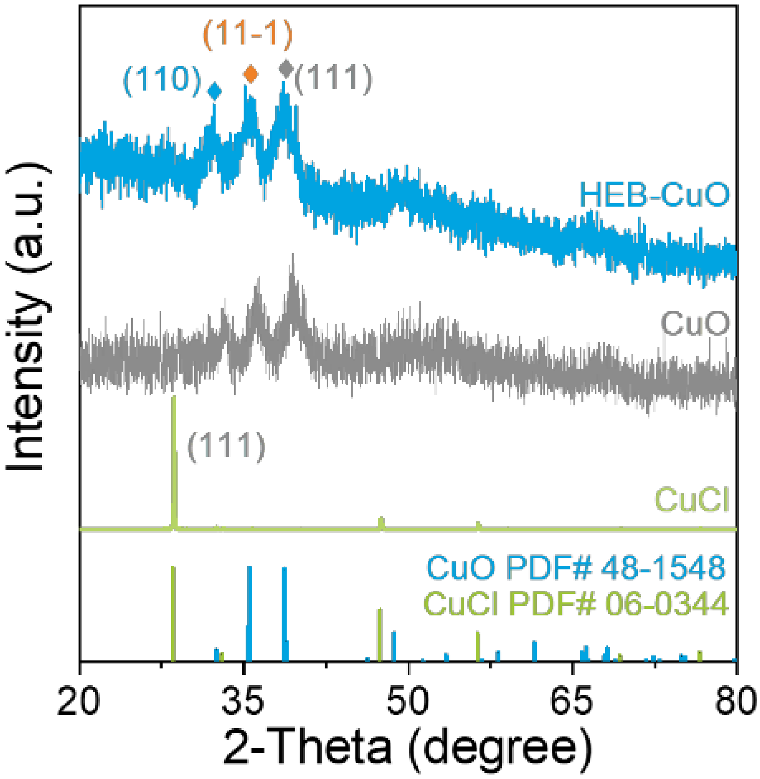

Fig. S2 XRD pattern of HEB-CuO, CuO and CuCl

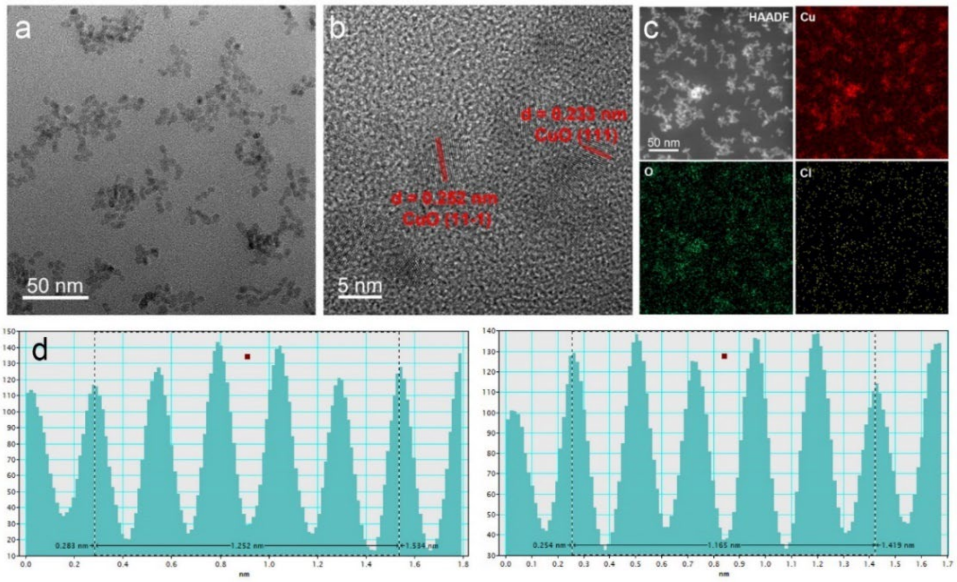

Fig. S3 Characterization of the morphology of CuO. (**a**) TEM, (**b**) HAADF-STEM, (**c**) EDS elemental mapping and (**d**) the corresponding intensity profile along the parallel lines as shown in (**b**)

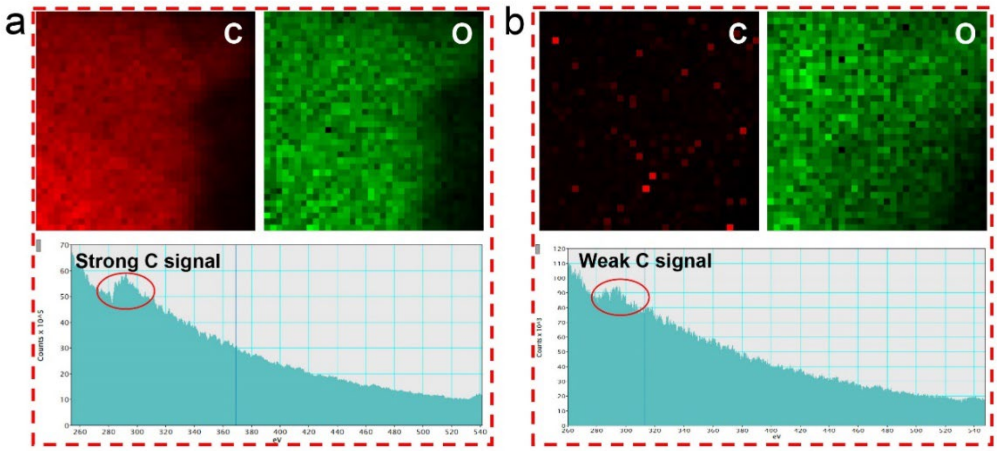

Fig. S4 EELS mapping and spectra of different catalysts. (**a**) HEB-CuO. (**b**) CuO

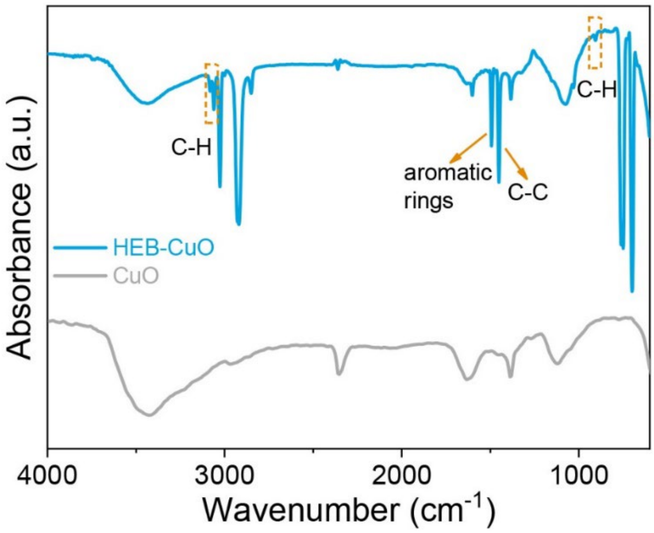

Fig. S5 FT-IR spectrum of HEB-CuO and CuO

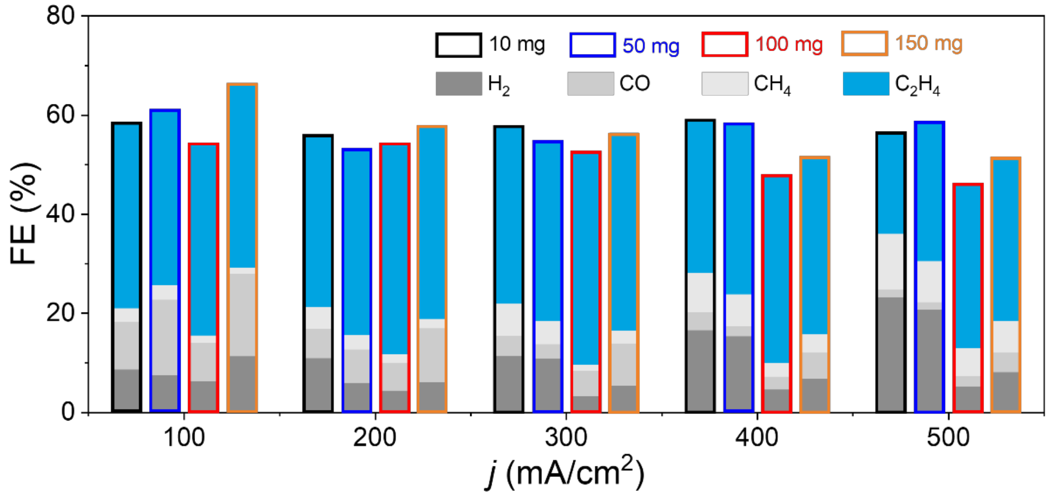

Fig. S6 FE of HEB-CuO with different contents of HEB-TMS precursor under different current density in 1M KCl. The feed amounts of the HEB-TMS precursor (100 mg) were confirmed to be the optimal ratio with a lower FE of H_2 and C_1 and good commercial feasibility

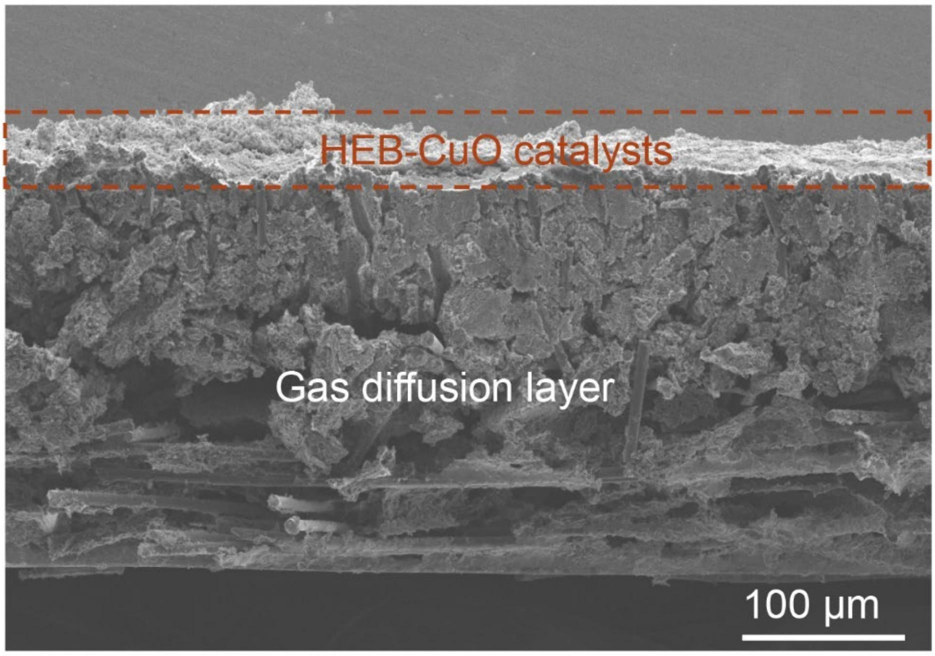

Fig. S7 Cross-section SEM images of HEB-CuO

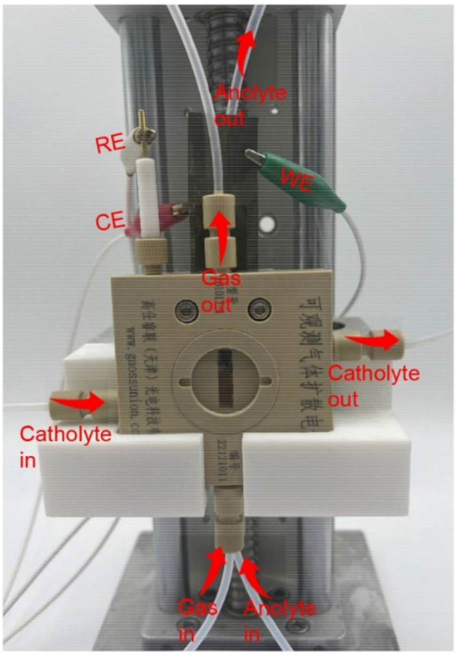

Fig. S8 Schematic illustration of the flow cell for CO_2RR tests

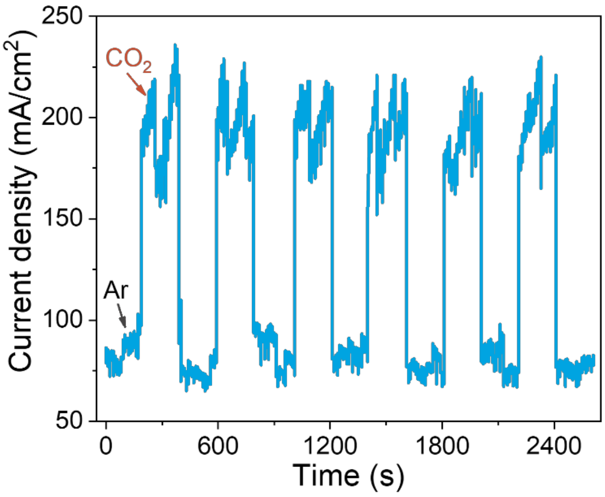

Fig. S9 Response curves of HEB-CuO catalyst in CO_2 and Ar atmosphere

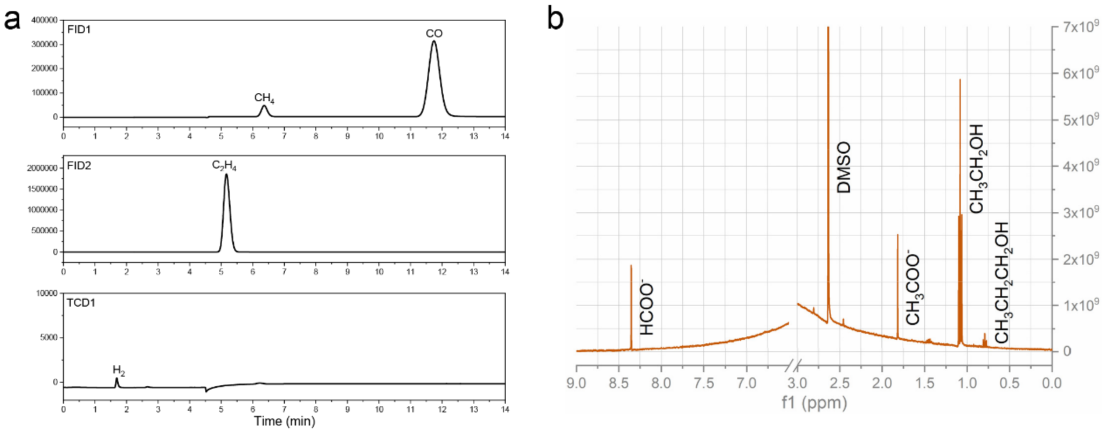

Fig. S10 GC spectroscopy and 1H NMR spectroscopy results. (**a**) Gas product spectra corresponding to FID1, FID2 and TCD detectors at 300 mA/cm^2. (**b**) Liquid phase product at 300 mA/cm^2

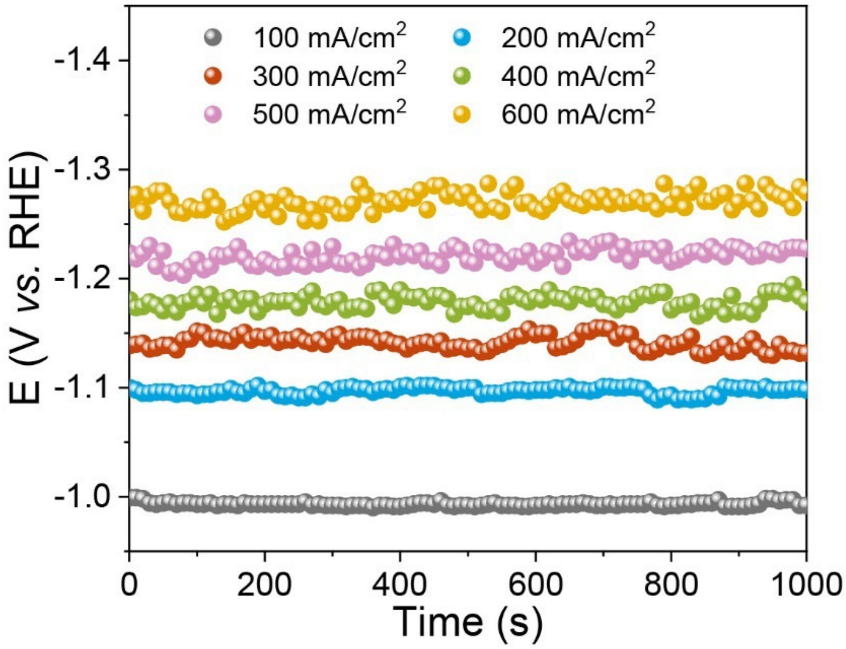

Fig. S11 Current densities for HEB-CuO over 1000 s of reaction at each given potential

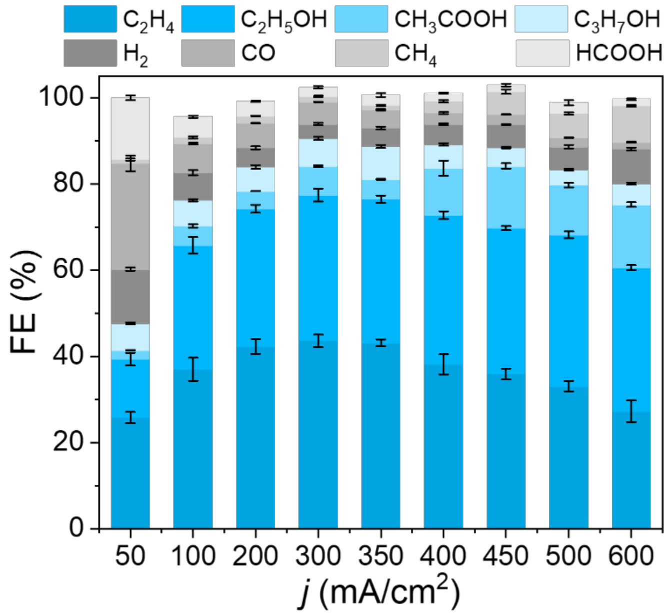

Fig. S12 FE of HEB-CuO under different current density in 1M KCl, which exhibiting 88.62% and 88.13% FE_{C2+} at 300 and 350 mA/cm^2

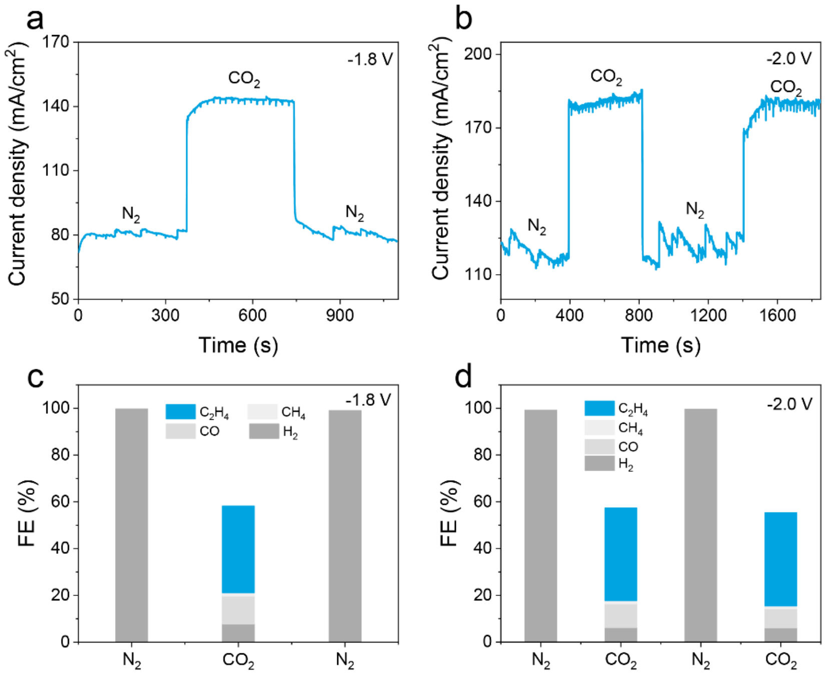

Fig. S13 Response curves of HEB-CuO catalyst in N_2 and CO_2 atmosphere and the corresponding FE of HEB-CuO under different atmosphere in 1M KCl at **a, c** -1.8 V, **c, d** -2.0 V (*vs.* Ag/AgCl). Under an N_2 atmosphere, the product is nearly 100% H_2; when the gas changes to CO_2, the gaseous products include H_2, CO, CH_4, and C_2H_4, which shows that the generated C_{2+} products all come from CO_2

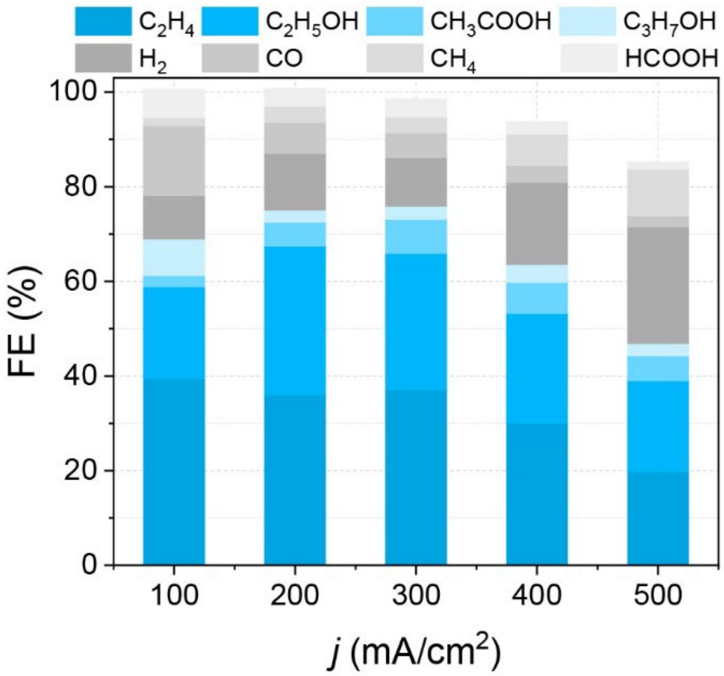

Fig. S14 FE of CuO under different current density in 1M KCl

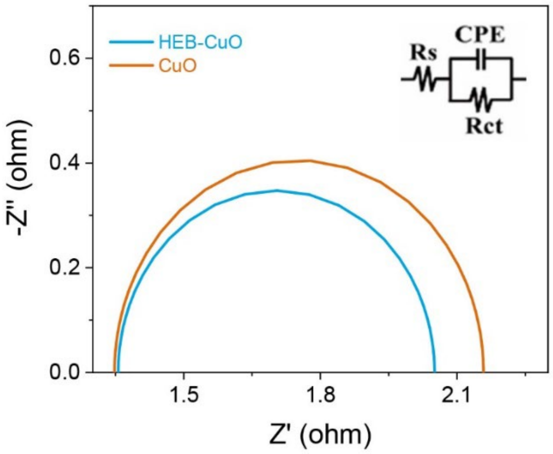

Fig. S15 EIS spectra of HEB-CuO and CuO. The frequency range is 0.1-10^5 Hz

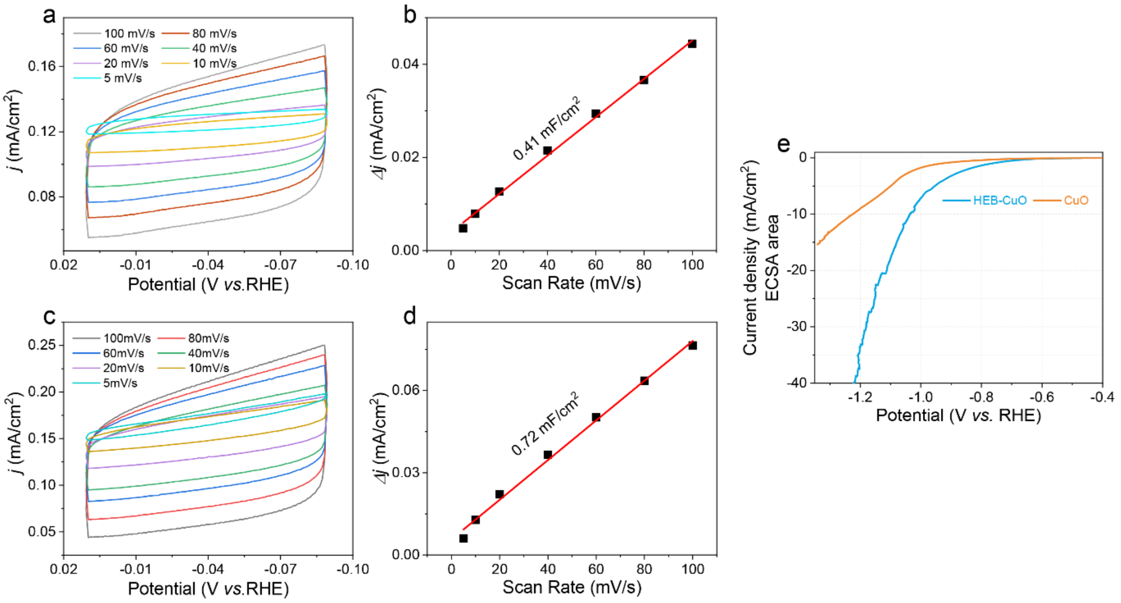

Fig. S16 The cyclic voltammetry profiles and electrical double-layer capacitances. (**a, b**) HEB-CuO. (**c, d**) CuO. **(e)** ECSA normalized LSV curves of HEB-CuO and CuO NPs [S7]

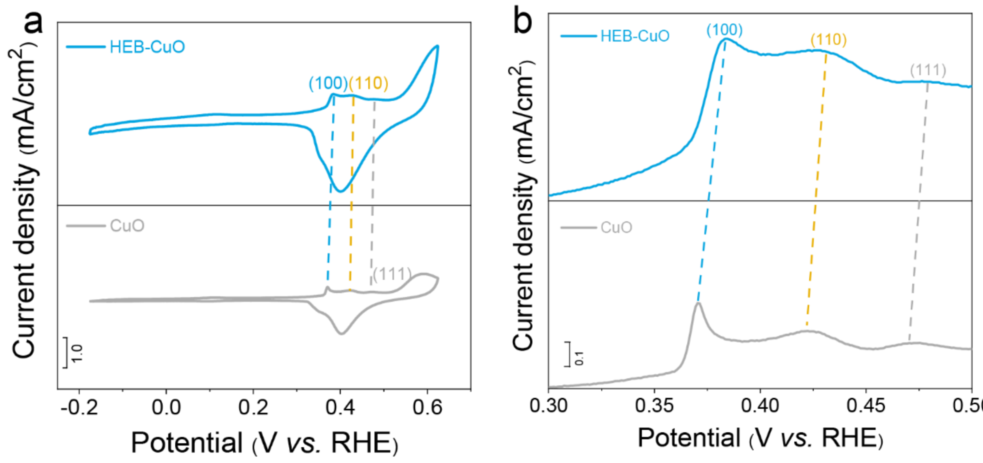

Fig. S17 Surface analysis of the HEB-CuO and CuO catalysts. (**a**) CV curve of catalysts in N_2-saturated 1 M KCl. (**b**) Fitted OH^- adsorption peaks. The CV curves show OH^- adsorption peaks at ~0.38, ~0.43 and ~0.48 V (vs. RHE) for HEB-CuO and CuO, corresponding to the OH^- adsorption on the Cu(100), Cu(110) and Cu(111) surfaces

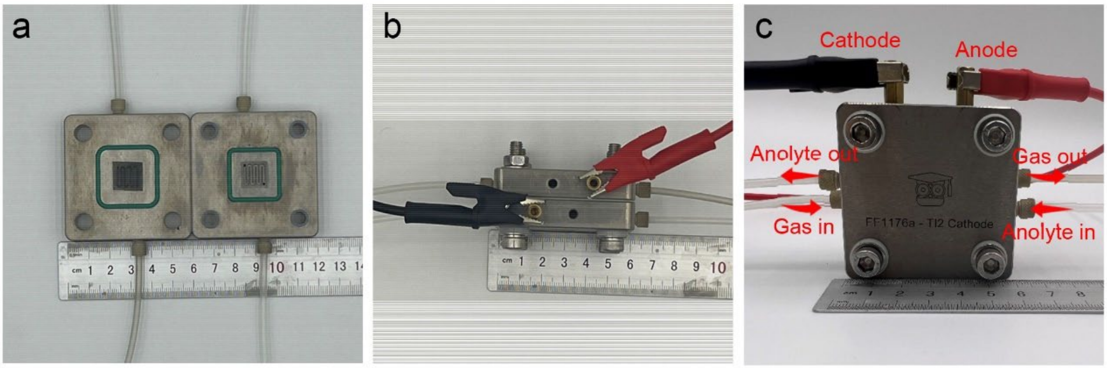

Fig. S18 Schematic illustration of the MEA for CO_2RR tests

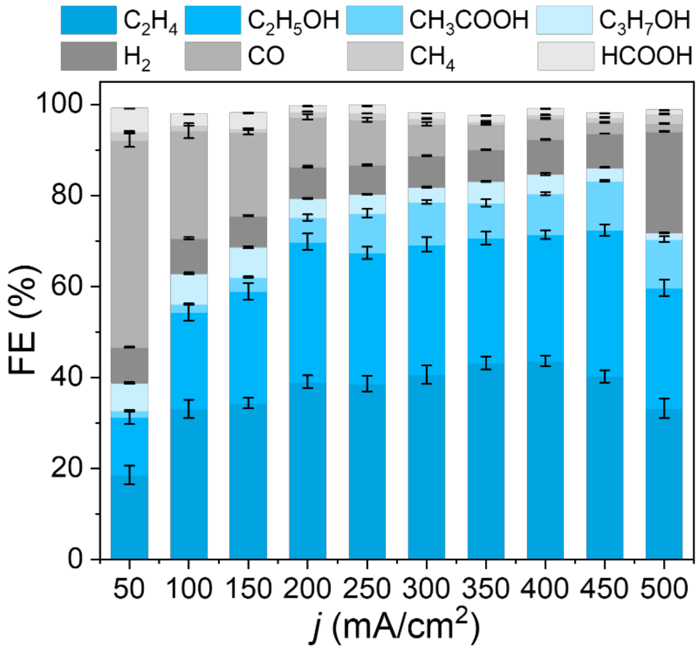

Fig. S19 CO_2RR performances of HEB-CuO in MEA

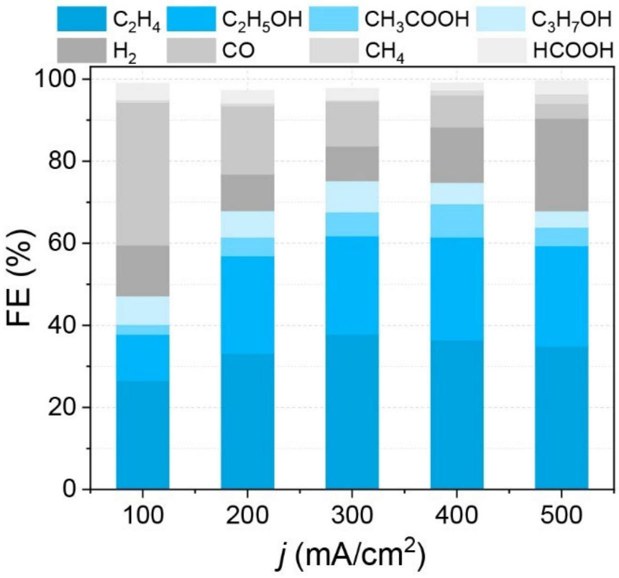

Fig. S20 CO_2RR performances of CuO in MEA

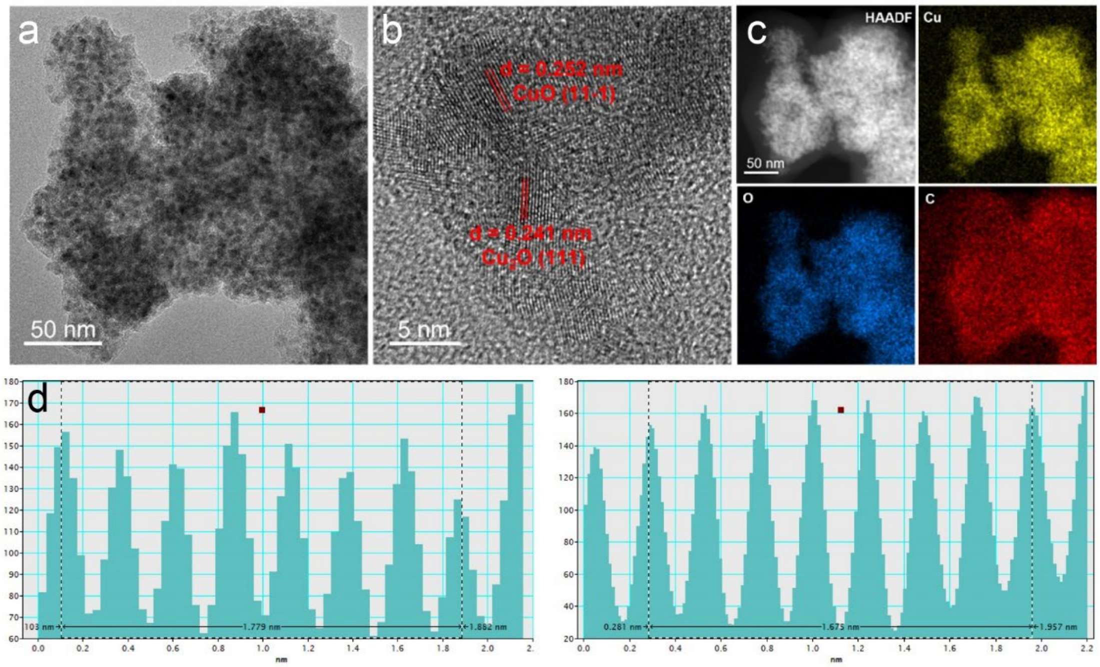

Fig. S21 Characterization of the morphology of HEB-CuO after CO_2RR tests. (**a**) TEM, (**b**) HAADF-STEM, (**c**) EDS elemental mapping and (**d**) the corresponding intensity profile along the parallel lines as shown in (**b**)

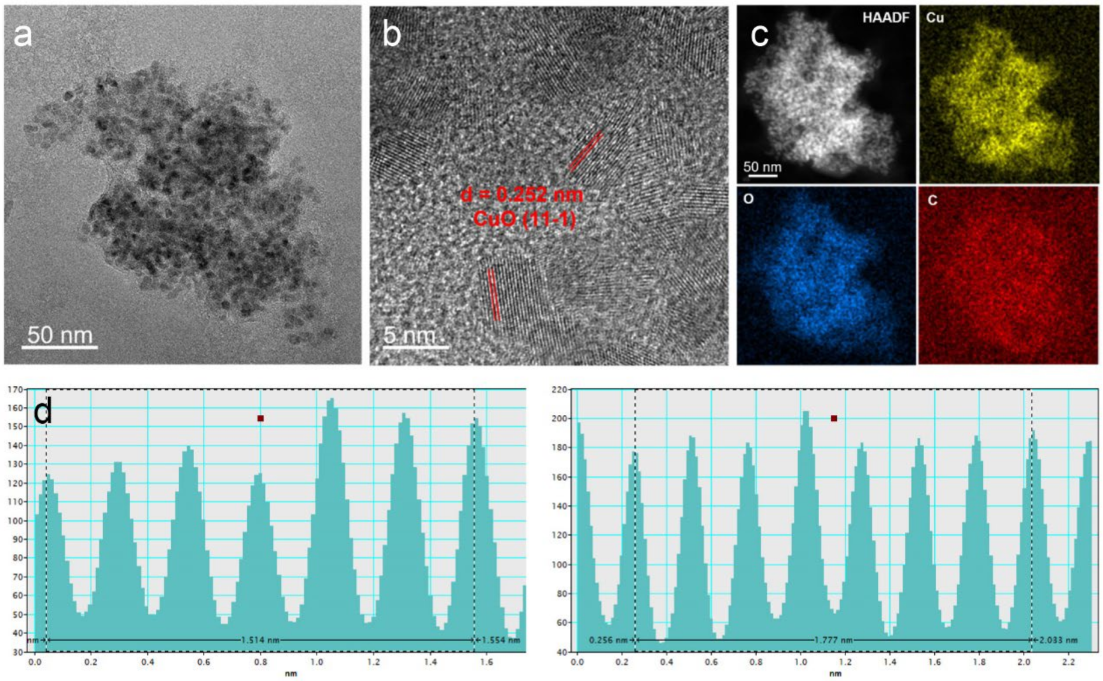

Fig. S22 Characterization of the morphology of CuO after CO_2RR tests. (**a**) TEM, (**b**) HAADF-STEM, (**c**) EDS elemental mapping and (**d**) the corresponding intensity profile along the parallel lines as shown in (**b**)

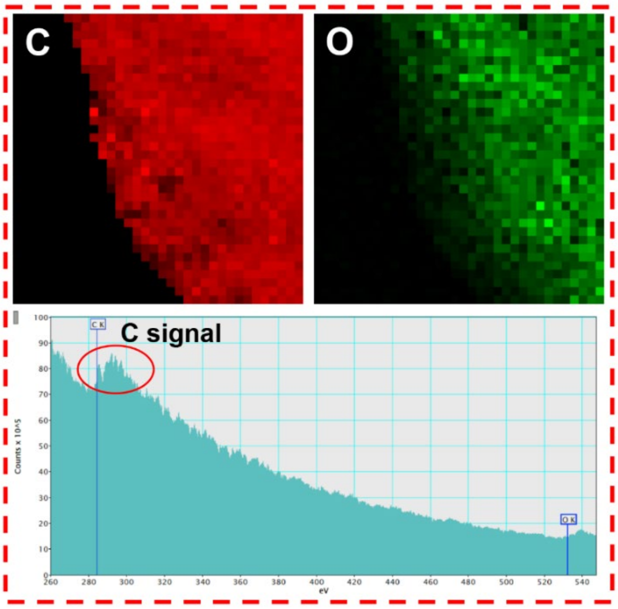

Fig. S23 EELS mapping and spectra of HEB-CuO after CO_2RR tests

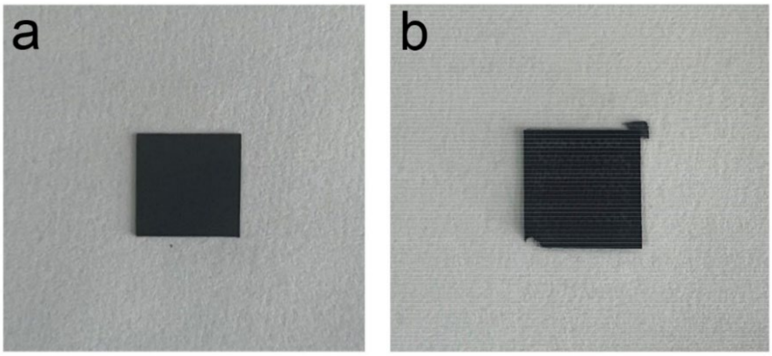

Fig. S24 Digital images of HEB-CuO electrode. (**a**) Before reaction. (**b**) After reaction

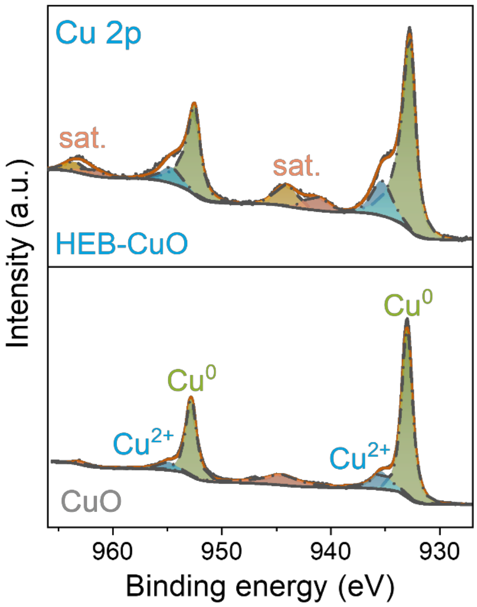

Fig. S25 XPS analysis of HEB-CuO and CuO after CO_2RR tests. Compared to before reaction, Cu(II) was reduced to Cu(0)

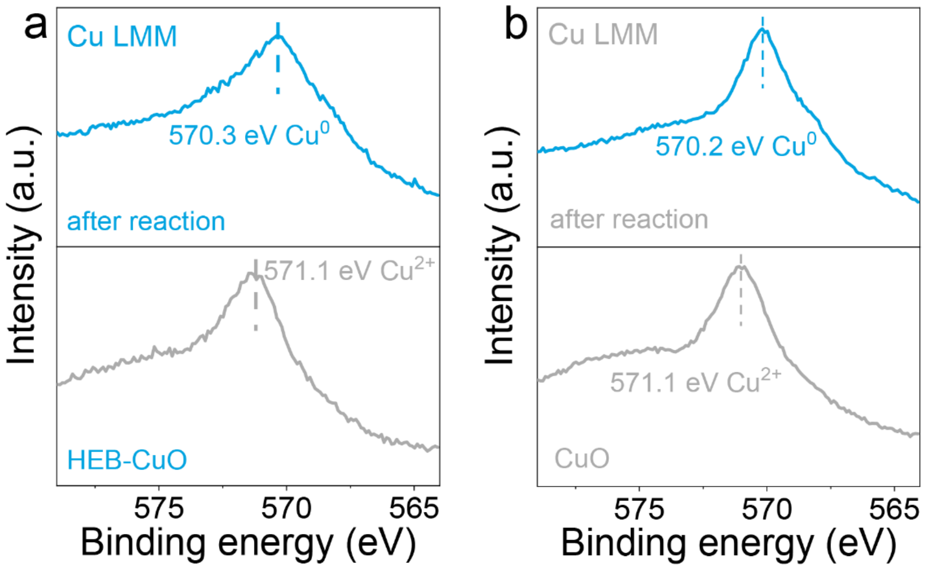

Fig. S26 Cu LMM of (**a**) HEB-CuO and (**b**) CuO. The Cu LMM XPS spectra showed a peak at ~571.1 eV, corresponding to the Cu^{2+}. After CO_2RR tests, a peak at ~570.3 eV, corresponding to the Cu^0 [S8]

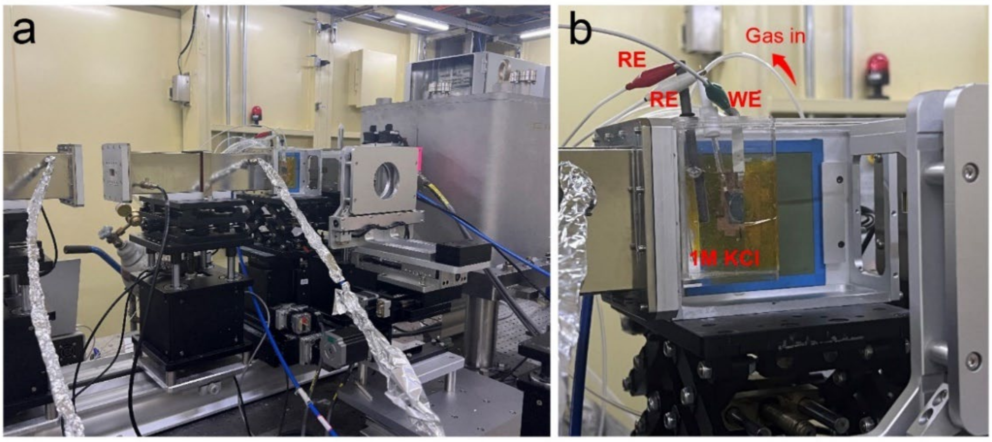

Fig. S27 Photograph of the electrochemical cell for in situ XAS measurements

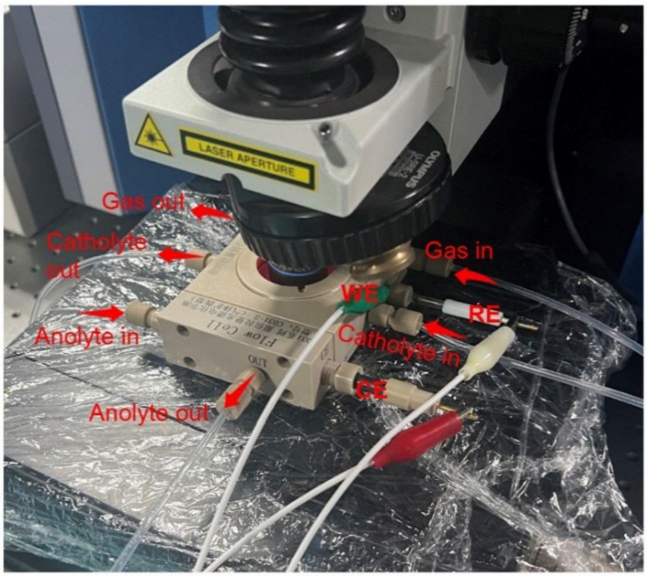

Fig. S28 Photograph of the electrochemical cell for *in-situ* Raman spectroscopy

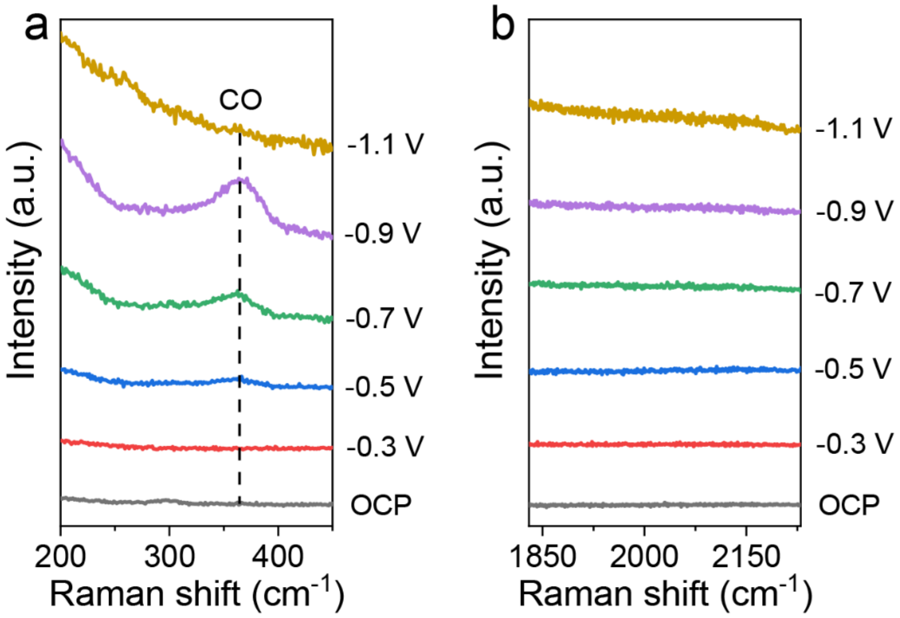

Fig. S29 *In-situ* Raman spectra at different ranges of the CuO catalyst. (**a**) In the range of 200-450 cm^{-1}, Cu-CO signal slowly appeared near 360 cm^{-1} and quickly disappeared. (**b**) In the range of 1830-2230 cm^{-1}. Neither signal was found in CuO

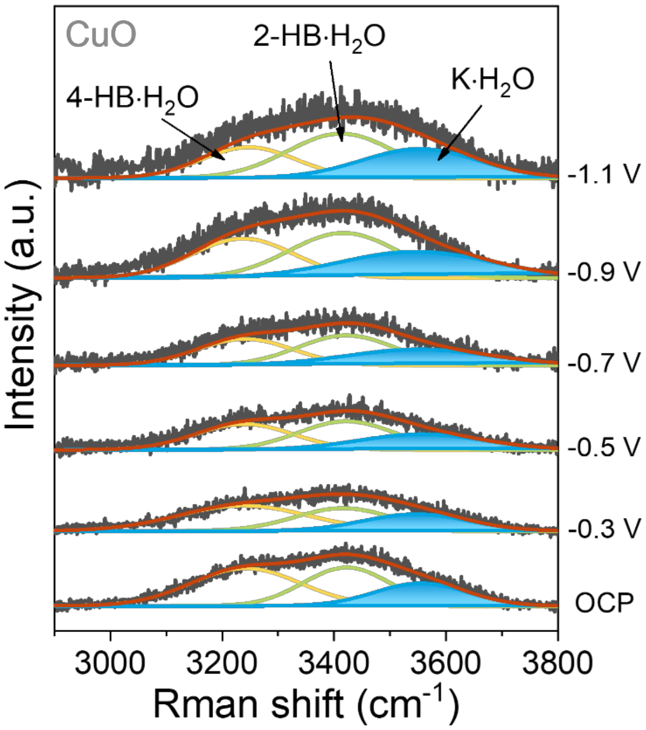

Fig. S30 *In-situ* Raman spectra of the interfacial water structure on CuO from OCP to -1.1 V

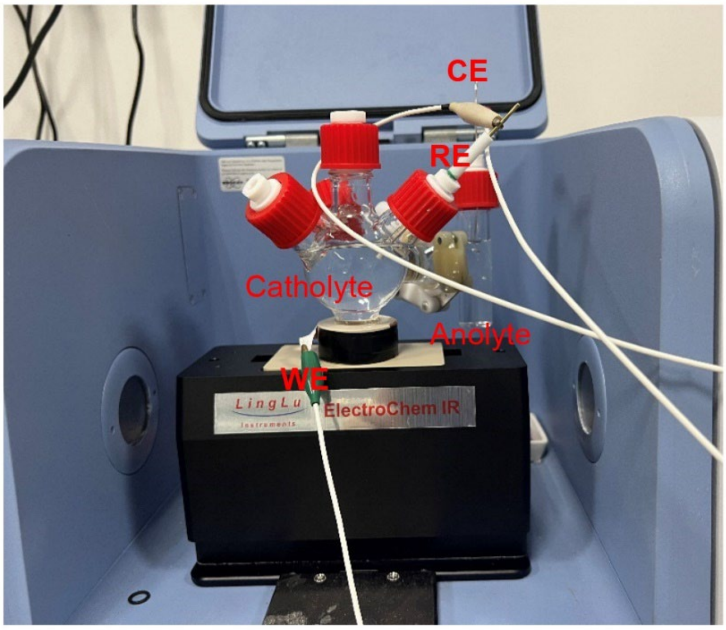

Fig. S31 Photograph of the electrochemical cell for *in-situ* ATR-SEIRS measurements

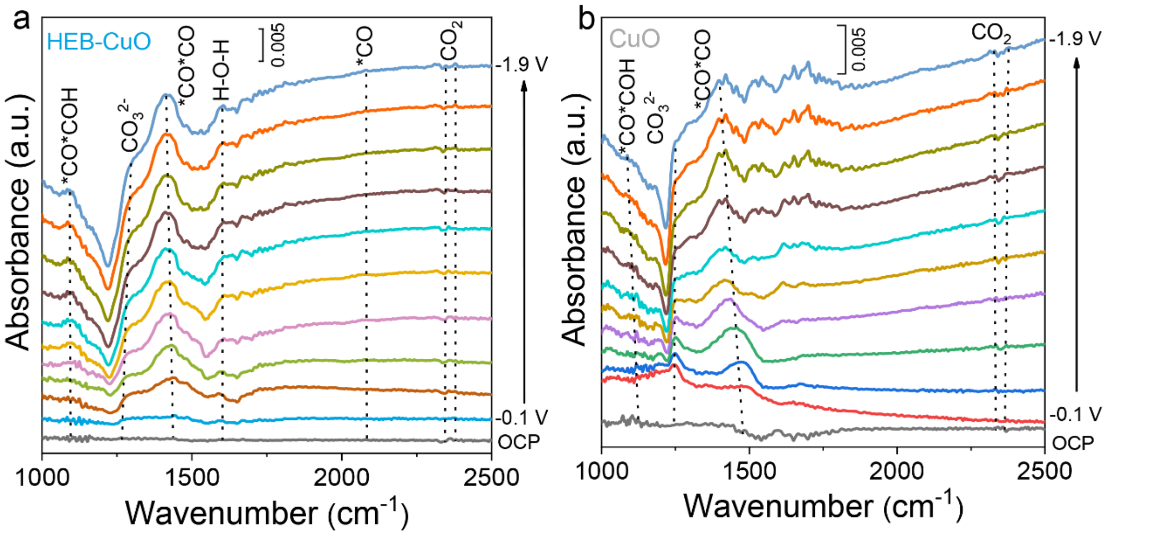

Fig. S32 *In-situ* ATR-SEIRS recorded at various applied potentials for HEB-CuO and CuO. The characteristic peak signal of HEB-CuO (**a**) and CuO (**b**) is consistent, but the overall signal of CuO is relatively weak

Table S1 CO_2RR performance of HEB-CuO catalyst with 1 M KCl in flow cell

Catalyst	J_{total} (mA/cm^2)	J_{C2+} (mA/cm^2)	FE (%)								
			C_2H_4	C_2H_5OH	CH_3COOH	C_3H_7OH	H_2	CO	CH_4	HCOOH	C_{2+}
HEB-CuO	50	23.86±0.32	25.87±1.27	13.55±1.47	2.02±0.04	6.27±0.23	12.58±0.34	24.56±1.83	0.85±0.21	14.31±0.55	47.71±0.62
	100	78.95±2.12	38.56±2.64	29.97±1.93	4.49±0.43	5.93±0.27	6.41±0.59	7.69±0.21	1.47±0.3	5.79±0.26	78.95±2.12
	200	167.96±2.56	42.37±1.74	31.91±0.95	4.09±0.05	5.61±0.42	4.42±0.35	5.72±0.04	1.68±0.08	3.38±0.14	83.98±1.28
	300	265.87±8.1	42.67±1.44	32.75±1.5	6.75±0.21	6.45±0.38	3.31±0.29	5.13±0.06	1.28±0.16	2.07±0.26	88.62±2.7
	350	308.45±4.84	42.83±0.78	33.08±0.88	4.57±0.09	7.64±0.38	4.27±0.3	4.25±0.14	1.04±0.14	2.38±0.43	88.13±1.38
	400	350.75±7.35	37.71±2.3	33.60±0.94	10.86±1.76	5.52±0.3	4.67±0.18	2.64±0.23	2.73±0.31	1.83±0.13	87.69±1.84
	450	386.89±4.4	35.48±1.25	33.81±0.5	12.40±0.64	4.28±0.16	5.32±0.12	2.37±0.08	5.30±0.5	1.49±0.32	85.98±0.98
	500	416.75±1.25	33.09±1.23	35.14±0.79	11.56±0.53	3.57±0.19	5.28±0.39	2.16±0.08	5.58±0.28	2.53±0.58	83.35±0.25
	600	480.50±12.02	27.33±2.52	33.32±0.56	14.62±0.56	4.82±0.25	8.04±0.33	1.59±0.11	8.45±0.24	1.46±0.21	80.08±2.0

Table S2 CO_2RR performance of HEB-CuO catalyst in 1 cm^2 MEA

Catalyst	J_{total} (mA/cm^2)	J_{C2+} (mA/cm^2)	FE (%)								
			C_2H_4	C_2H_5OH	CH_3COOH	C_3H_7OH	H_2	CO	CH_4	HCOOH	C_{2+}
HEB-CuO	50	19.41±0.27	18.59±2.04	12.69±1.53	1.40±0.16	6.13±0.16	7.86±0.09	45.45±1.46	1.88±0.18	5.10±0.03	38.82±0.53
	100	62.93±0.34	33.11±2.01	21.29±1.87	1.71±0.07	6.82±0.18	7.66±0.22	23.62±1.65	1.19±0.04	2.43±0.04	62.93±0.34
	150	102.98±1.14	34.40±1.13	24.57±1.8	3.13±0.13	6.56±0.23	6.90±0.12	18.33±0.52	0.90±0.04	3.34±0.12	68.65±0.76
	200	158.77±2.18	39.07±1.38	30.78±1.82	5.30±0.66	4.23±0.12	6.96±0.19	10.95±0.58	1.15±0.02	1.20±0.03	79.38±1.09
	250	200.72±2.65	38.63±1.74	28.75±1.33	8.74±0.93	4.16±0.12	6.43±0.15	9.97±0.44	1.41±0.03	1.62±0.11	80.29±1.06
	300	245.42±1.41	40.67±2.06	28.56±1.61	9.33±0.41	3.24±0.17	6.95±0.08	6.98±0.38	1.19±0.09	1.08±0.05	81.81±0.47
	350	290.82±2.3	43.20±1.4	27.47±1.44	7.71±0.81	4.72±0.11	7.00±0.1	5.58±0.15	0.61±0.14	1.28±0.03	83.09±0.66
	400	338.84±2.82	43.68±1.15	27.72±0.95	9.05±0.34	4.26±0.22	7.58±0.12	4.67±0.13	0.78±0.02	1.31±0.03	84.71±0.71
	450	387.64±0.5	40.28±1.35	32.12±1.28	10.86±0.14	2.87±0.09	7.45±0.02	2.52±0.14	1.08±0.07	0.86±0.04	86.14±0.11
	500	358.97±1.58	33.25±2.13	26.46±1.79	10.66±0.62	1.42±0.05	22.29±0.14	1.74±0.12	2.13±0.1	0.81±0.04	71.79±0.32

Table S3 Comparison of CO_2RR-to-C_{2+} products over various reported Cu-based electrocatalysts

**Electrocatalyst**	**Electrolyte**	**FE_{C2+} (%)**	**J_{C2+} (mA/cm^2)**	**Stability (h)**	**Cell**	**Refs.**
HEB-CuO	1 M KCl	88.4	265.2	10	flow cell	**This work**
	0.1 M KOH	86.1	387.4	50	MEA	
Solution-grown Cu NPs	1 M KOH	26	150	4	flow cell	[S9]
Ce(OH)$_x$/Cu/PTFE	1 M KOH	80.3	240.9	6	flow cell	[S10]
Cu nanosheets	2 M KOH	69	144.9	3	flow cell	[S11]
LSTr-Cu	1 M KOH	55	204.8	1.7	flow cell	[S12]
Cu−S motif on HKUST-1	1 M KOH	88.4	228.8	8	flow cell	[S13]
Cu-SiO$_x$	0.1 M $KHCO_3$	81	267.9	50	MEA	[S14]
Cu-KOH	1 M KOH	78.7	221	6	MEA	[S15]
Defect-rich-Cu	0.1 M $KHCO_3$	60	120	30	MEA	[S16]
Dual-phase Cu	3 M KCl	80	322	45	MEA	[S17]
Cu(100)/Cu(111)	1 M $KHCO_3$	74.9	224.7	50	MEA	[S18]

* The electrolyte in Flow cell is the Catholyte, and in MEA is the anolyte

Supplementary References

[S1] K.P. Kuhl, E.R. Cave, D.N. Abram, T.F. Jaramillo, New insights into the electrochemical reduction of carbon dioxide on metallic copper surfaces. Energy Environ. Sci. **5**, 7050–7059 (2012). https://doi.org/10.1039/C2EE21234J

[S2] S. Plimpton, Fast parallel algorithms for short-range molecular dynamics. J. Comput. Phys. **117**, 1–19 (1995). https://doi.org/10.1006/jcph.1995.1039

[S3] G. Kresse, J. Furthmüller, Efficient iterative schemes for *ab initio* total-energy calculations using a plane-wave basis set. Phys. Rev. B Condens. Matter **54**, 11169–11186 (1996). https://doi.org/10.1103/physrevb.54.11169

[S4] D.C. Rapaport, The Art of Molecular Dynamics Simulation. Cambridge University Press, (2004).

[S5] L. Li, Z. Xia, Y. Yang, Molecular dynamics simulation of nanoimprinting under a high-frequency vibration perturbation. J. Comput. Theor. Nanosci. **9**, 35–40 (2012). https://doi.org/10.1166/jctn.2012.1993

[S6] W.G. Hoover, Canonical dynamics: Equilibrium phase-space distributions. Phys. Rev. A Gen. Phys. **31**, 1695–1697 (1985). https://doi.org/10.1103/physreva.31.1695

[S7] D. Voiry, M. Chhowalla, Y. Gogotsi, N.A. Kotov, Y. Li et al., Best practices for reporting electrocatalytic performance of nanomaterials. ACS Nano **12**, 9635–9638 (2018). https://doi.org/10.1021/acsnano.8b07700

[S8] C. Liu, X.-D. Zhang, J.-M. Huang, M.-X. Guan, M. Xu et al., *In situ* reconstruction of Cu–N coordinated MOFs to generate dispersive Cu/Cu_2O nanoclusters for selective electroreduction of CO_2 to C_2H_4. ACS Catal. **12**, 15230–15240 (2022). https://doi.org/10.1021/acscatal.2c04275

[S9] S. Ma, M. Sadakiyo, R. Luo, M. Heima, M. Yamauchi et al., One-step electrosynthesis of ethylene and ethanol from CO_2 in an alkaline electrolyzer. J. Power Sources **301**, 219–228 (2016). https://doi.org/10.1016/j.jpowsour.2015.09.124

[S10] M. Luo, Z. Wang, Y.C. Li, J. Li, F. Li et al., Hydroxide promotes carbon dioxide electroreduction to ethanol on copper via tuning of adsorbed hydrogen. Nat. Commun. **10**, 5814 (2019). https://doi.org/10.1038/s41467-019-13833-8

[S11] W. Luc, X. Fu, J. Shi, J.-J. Lv, M. Jouny et al., Two-dimensional copper nanosheets for electrochemical reduction of carbon monoxide to acetate. Nat. Catal. **2**, 423–430 (2019). https://doi.org/10.1038/s41929-019-0269-8

[S12] Y. Li, F. Liu, Z. Chen, L. Shi, Z. Zhang et al., Perovskite-socketed sub-3 nm copper for enhanced CO_2 electroreduction to C_2. Adv. Mater. **34**, e2206002 (2022). https://doi.org/10.1002/adma.202206002

[S13] C.F. Wen, M. Zhou, P.F. Liu, Y. Liu, X. Wu et al., Highly ethylene-selective electrocatalytic CO_2 reduction enabled by isolated Cu-S motifs in metal-organic framework based precatalysts. Angew. Chem. Int. Ed. **61**, e202111700 (2022). https://doi.org/10.1002/anie.202111700

[S14] J. Li, A. Ozden, M. Wan, Y. Hu, F. Li et al., Silica-copper catalyst interfaces enable carbon-carbon coupling towards ethylene electrosynthesis. Nat. Commun. **12**, 2808 (2021). https://doi.org/10.1038/s41467-021-23023-0

[S15] D. Higgins, C. Hahn, C. Xiang, T.F. Jaramillo, A.Z. Weber Gas-diffusion electrodes for carbon dioxide reduction: a new paradigm. ACS Energy Lett. **4**, 317–324 (2019). https://doi.org/10.1021/acsenergylett.8b02035

[S16] Gu Z., Shen H., Chen Z., Yang Y., Yang C. et al., Efficient electrocatalytic CO_2 reduction to C^{2+} alcohols at defect-site-rich Cu surface. Joule **5**, 429–440 (2021). https://doi.org/10.1016/j.joule.2020.12.011

[S17] P.-P. Yang, X.-L. Zhang, P. Liu, D.J. Kelly, Z.-Z. Niu et al., Highly enhanced chloride adsorption mediates efficient neutral CO_2 electroreduction over a dual-phase copper

catalyst. J. Am. Chem. Soc. **145**, 8714–8725 (2023). https://doi.org/10.1021/jacs.3c02130

[S18] Z.-Z. Wu, X.-L. Zhang, Z.-Z. Niu, F.-Y. Gao, P.-P. Yang et al., Identification of Cu(100)/Cu(111) interfaces as superior active sites for CO dimerization during CO_2 electroreduction. J. Am. Chem. Soc. **144**, 259–269 (2022). https://doi.org/10.1021/jacs.1c09508
